# The effect of different levels of pre-damage loading on the strength and structural behavior of CFRP strengthened R.C. beams: Experimental and analytical investigation

**DOI:** 10.1371/journal.pone.0261290

**Published:** 2021-12-30

**Authors:** Brwa Hamah Saeed Hamah-Ali, Mohamed Raouf Abdul Qadir

**Affiliations:** 1 Ph.D. Student at Civil Engineering Department, College of Engineering, University of Sulaimani, Sulaymaniyah, Iraq; 2 Professor at Civil Engineering Department, College of Engineering, University of Sulaimani, Sulaymaniyah, Iraq; University of Vigo, SPAIN

## Abstract

In order to investigate the effect of pre-loading damage on the structural performance of Carbon Fiber Reinforced Polymer (CFRP) strengthened Reinforced Concrete (R.C.) beams, experimental and Finite Element Modelling (FEM) investigation was carried out on six R.C. beams. Five of the R.C. beams were damaged up to different levels of strain in the main steel bars before Flexure CFRP strengthening. One of the R.C. beams loaded up to failure and was kept as a control beam for comparison. The experimental results showed that the failure mode of the CFRP strengthened specimen was controlled by CFRP debonding followed by concrete crushing; however, the control beam failed in concrete crushing after yielding the steel bars, which is a ductile failure. The CFRP sheet increases the strength and initial stiffness of the R.C. beams and reduces ductility and toughness. Also, CFRP application increases the first crack and yielding steel bars load by 87.4% and 34.4%, respectively. Furthermore, the pre-damage level does not influence the strength and ductility of the strengthened R.C. beams except for the highest damage levels, which experienced a slight decrease in load capacity and ductility. However, the initial stiffness decreases with increasing pre-damage levels by 40%.

Design guideline ACI 440.2R (2004) predicts the ultimate load capacity marvelously for externally bonded Fiber-Reinforced Polymer (FRP) beams compared to the experimental maximum load capacity. The excellent agreement between experimental and FEM results indicates that the constitutive models used for concrete and reinforcement and the cohesive interface model can well capture fracture behavior. However, The FEM analysis predicts the beam to be slightly stiffer and more robust, probably because of the assumed perfect bond between concrete and reinforcement. The developed FEM can be used for further parametric study.

## 1. Introduction

Many R.C. structures are damaged. Most of them are suffering from various deteriorations such as cracks and concrete spalling. Many factors are at the origin of these deteriorations, such as aging, corrosion of steel, earthquake, environmental effects, and accidental impacts on the structure [1, 2].

Nowadays, it is necessary to find repair techniques suitable for low costs and fast processing time. Externally bonded FRP has emerged as a new structural strengthening technology in response to the increasing need for repair and strengthening of reinforced concrete structures because of their high tensile strength, lightweight, resistance to corrosion, high durability, and ease of installation [1, 3].

The FRP is characterized by high-strength fibers embedded in polymer resin. The most common type of FRP in the industry is made with carbon, aramid, or glass fibers. Repairing beam structures by externally bonded FRP composites consists of adhering FRP laminates at the tensile face of the beam. Among these types of FRP, the application of CFRP to strengthen and repair the concrete beams has received the most attention from the research community [1–3].

[4] reviewed the test methods of studying the FRP-concrete bond characteristics. The primary techniques and modifications used by different researchers were given and reviewed. The advantages and disadvantages of each test technique were discussed, followed by some perspectives and the need for future study. Finally, a new testing arrangement for the double shear lap test was presented, along with its advantages over the current technique. The authors summarized that following the inspection of the concrete surface and calculation of the parameters required for strengthening, a variety of established in situ techniques of reinforcing structures with FRP sheets exist. Nonetheless, early FRP debonding is still a key impediment to the successful application of FRP. This might be due to unfavorable environmental conditions that the reinforced constructions may be subjected to. Premature debonding may also occur as a result of unanticipated changes in the load pattern that were not accounted for during the strengthening process. It was recommended that this type of early debonding can be prevented by providing either surface treatment of concrete prisms or other types of anchoring systems. The preparation of concrete specimens, which is responsible for the strength qualities of concrete, is critical in the FRP-concrete bond. Bond strength is more closely connected to surface tensile strength and coarse aggregate content in concrete than other strength parameters. Again, the influence of surface roughness on bond capacity is well understood to result in more intact mechanical interlocking at the contact.

[5] carried out an experimental study and 2- and 3-D numerical simulations to examine the performance of FRP- concrete bond-slip behavior using the double shear test. An improved clamping double shear test was compared with the traditional double shear test method. The test variable was the FRP bond length. The test results showed that the failure mode of the specimens was the FRP debonding along the FRP-Epoxy interface and the adjacent concrete substrate layers. Furthermore, in general, bond length was shown to have little influence on the interaction between the concrete and CFRP specimens examined as long as the bond length was more than the effective bond length. The 3-D Finite Element (FE) models provided more similar findings to the experiments than the 2-D FE models, particularly in terms of ultimate load. The inaccuracies in the 2-D models might be attributed to simplifying assumptions and the use of an empirical model to compensate for not utilizing the same width of CFRP as concrete width. The authors validated the experimental and numerical simulations using the bond-slip models presented in the literature. There are large discrepancies in values across the various literature models, indicating subjectivity in the prediction of interaction between concrete and FRP in general. The causes for these disparities in literature predictions are related to variances in the factors examined by respective models. For example, while some examine the FRP-to-concrete width ratio, others consider concrete compressive strength, and still others consider concrete tensile strength with various assumptions.

[6] also carried out an experimental investigation and FEM analysis in order to study the shear behavior and debonding characteristics of initially ductile R.C. beams (failing in flexure when unstrengthened) at the verge of transitioning to a more brittle shear failure upon strengthening using external flexural CFRP. A total of seven beams–two control (unstrengthened) and five flexural CFRP‐ strengthened specimens–were tested. The test results concluded that despite the lack of external shear strengthening, the external flexural CFRP strengthening contributes significantly to shear capacity. Increases in the CFRP area improve this capacity, but the rate of improvement decreases when the theoretical flexural strength approaches the shear capacity. Moreover, the location of the major shear failure crack shifts away from the loaded point and towards the CFRP cutoff point as the CFRP area ratio and/or stiffness increase (theoretical flexural capacity exceeds the theoretical shear capacity). The CFRP cutoff point is the ultimate location of such a crack (a perfect plate end shear crack). The shear crack induced CFRP debonding increases with increase in the CFRP bending stiffness as the CFRP dowel action becomes more pronounced. The CFRP delamination can be taken into account by the numerical simulation, which uses cohesive zone elements.

[7] have conducted extensive experimental and numerical studies to understand the effect of concrete heterogeneity on the FRP-concrete bond behavior. FRP-concrete and FRP-mortar bonded joints were tested under a four-point bending setup. The crack initiation and propagation process with interfacial debonding until failure was accurately captured by the Digital Image Correlation (DIC) technique. The results showed that the presence of coarse aggregates in the FRP-concrete joints leads to 19% higher bond strength, but much higher variations in the bond strength and the strain distribution across the width of FRP sheets, than the FRP-mortar joints. Also, It was found that the distribution of coarse aggregates significantly affected the overall load-deflection responses. The DIC study showed that there were two distinct failure modes in the beam test setup: the flexural-shear crack-induced FRP debonding and the brittle block splitting from the FRP bonding end. The size, shape, and orientation of coarse aggregates and their distribution play a significant role in the forming of two distinct final failure modes.

[8] developed typical bond-slip models of two specimen types; one containing fresh aggregate and the other consisting of steel slag aggregate substituting 30% of the maximum fresh aggregate size in the first concrete type in order to study the influence of coarse aggregate type on FRP-concrete bond behavior using the double shear test. The test results observed that substituting the fresh coarse aggregate with steel slag aggregate, although in a small amount, enhanced the bond-slip characteristics. The author concluded that due to the abrupt termination of the softening region of bond-slip behavior for the specimen containing steel slag, there is need for further research to consider several other parameters. The experimental bond-slip parameters determined in this study reflect the quality of the substrate aggregate. The so determined parameters can be used as input for numerical simulations in developing analytical bond models or substituting them into existing models to predict the bond behavior.

The performance of Externally Bonded (EB) FRP to concrete is heavily impacted by the environmental conditions to which it is subjected while in service. The two most prevalent environmental conditions that affect the bond behavior of EB-FRP are temperature and humidity [9]. [9] reviewed the experimental and computational approaches used to evaluate the effect of the hygrothermal (humidity and temperature) effects on the durability of the FRP-concrete bond, as well as on the performance of the bond under loading conditions. Based on the reviewed literature, the authors concluded that the mode of failure of the FRP–concrete bond largely depends on the size of the specimens, exposure conditions, surface condition of the FRP–concrete interface, method of conditioning, exposure duration, and methods of testing. Furthermore, it is being summarized that moisture initially provides an ambient curing condition for the adhesive, but in the long term, it deteriorates both the fiber and the adhesive, while temperature, at a value close to the glass transition, can change the behavior of the adhesive. The authors claimed that the use of nano-modified epoxy for FRP–concrete bonding seems to be a solution to FRP–concrete bond durability problems.

[10] carried out numerous double lap shear tests to study FRP-concrete bond performance under five weeks of exposure to several accelerated hygrothermal conditions, examining in the process the performance of two concrete types (with and without steel slag). Additionally, the material properties of epoxy adhesive and dry FRP fibers under the same hygrothermal conditions were investigated. The tests revealed that at low humidity (dry heat), FRP-concrete bond strength increased as temperature increased—a possible consequence of alteration in the epoxy glass transition temperature. The bond strength of concrete with steel slag was superior at all temperatures and low humidity. At low humidity, specimens kept near the glass transition temperature (60°C) of the epoxy ruptured at a lower strain than predicted by material tests. Specimens heated beyond the glass transition temperature (to 100°C) had the greatest bond capacity but failed within the concrete. The failure mode transitioned from substrate failure to adhesive-concrete interface failure under high humidity conditions where the specimens were continuously or periodically kept under water. Two-day alternating wet/dry cycles resulted in a 12% decline in bond strength. Bond capacity was not adversely impacted by hygrothermally cured epoxy (at 60°C), but this resulted in purely interfacial failures between adhesive and concrete with some partial substrate debonding. While the measured bond strength was similar to that observed under ambient conditions, the fracture energy much smaller. At low humidity (dry heat), the FRP dry fiber tensile stress increased as temperature increased, while epoxy coupons exhibited the opposite behavior. The presence of moisture at ambient temperature resulted in similar drops in mechanical properties in specimens of both epoxy and FRP materials. Local bond shear strength and, therefore, fracture energy was overestimated when the corresponding degraded material properties were not used to analyze the FRP-concrete bond exposed to hygrothermal conditions.

The most published work on damaged R.C. beams repaired by FRP dealt with repairing these structures damaged by the corrosion effect. However, several studies have been carried out on the retrofitting of the pre-damage R.C. beams due to overloading [11–13].

[14] carried out experimental work on six T-shaped full-scale R.C. beams. The R.C. beams were pre-damaged based on the crack width of 0.3mm without yielding the steel reinforcement. Furthermore, the damaged R.C. beams were repaired using carbon fiber reinforced polymer (E = 165 GPa and fu = 1000 MPa). After testing the R.C. beams using four-point static loading, the authors concluded that the damaged R.C. beams increased their ultimate capacity and stiffness by 34% and 65%, respectively, after strengthening compared to the control beams. In terms of ductility, all the repaired R.C. beams failed by debonding CFRP; thus, the ductility was decreased compared to the control beams. Moreover, in comparison to the control beams, strengthened beams exhibited a much lower increase in crack width. This is due to the increased CFRP tensile reinforcement.

[2] carried out an experimental investigation on six normal strength R.C. beams with the same reinforcement detailing and concrete strength. Three of the R.C. beams were tested as control beams, and they failed in flexure. The three others were pre-loaded damage beams up to 70% of the ultimate load of the control beams. In conclusion, the damage beams increased their load-carrying capacity by 17% when strengthened with a 100 mm width and 1.2 mm thick of the CFRP sheet in a single layer compared to the control beam. Furthermore, the first flexural cracks of the strengthened appear at a higher load.

[15] has reported the structural performance of initially cracked R.C. one-way slabs strengthened with different techniques. Initially, all the slabs loaded to (2/3) of their expected ultimate load capacity except for the control slab, which was loaded until failure. The pre-cracked R.C. slabs were repaired with five different techniques. The researchers used grout pouring S2, epoxy injection S3, ferrocement S4, CFRP strips S5, and section enlargement S6 as strengthening strategies. The R.C. slab S5 was strengthened using a 50mm wide CFRP strip externally bonded to the tension face of the R.C. slab. It was concluded that the ultimate load capacity of slab S5 showed a 77.4% higher ultimate load capacity than the control slab. The specimen reinforced with the carbon fiber strip shows no change in the initial stiffness compared to the control slab. With the loading increase, the stiffness decreases at a higher rate than specimens S1, S2, S3, and S4. Hence, the CFRP strip significantly affects the stiffness in the advanced stage of loading. Moreover, concrete compressive strain at mid-span at a distance of 25mm below the top fiber of R.C. slab specimens was recorded. All the slab specimens exhibit lower strain values compared to the control slab. The decrease in the concrete strain in slab S5 has been recorded at 65%. The failure mechanism in Slab S5 is characterized by the shearing of the concrete interface with the CFRP strip (relative slippage) associated with minor warnings compared to other slab specimens. The failure was sudden and occurred immediately after the peeling of the CFRP strips; this is due to the insufficient anchorage length of the CFRP strip. The strain measured before failure in the CFRP laminate is 60% of its yielding strain, which complies with peeling failure (and not rupture failure) observed in this specimen. The number of cracks observed at failure is more, but the width of the crack is smaller compared to other samples. The ductility performance for slab S5 was less than that of the control slab.

[2] investigated the flexural behavior of repaired R.C. beams using CFRP laminates and the contribution of CFRP laminates to restore the strength and rigidity of the restored beams. The foremost parameter of their work was the damage degree, which has been taken as (0%, 80%, 90%, and100%) of the ultimate load capacity of the control beam. The authors observed that repairing damaged R.C. beams with externally bonded CFRP laminates was successful for different degrees of damage. Therefore, this technique effectively restores the mechanical performance of cracked or damaged R.C. beams.

The failure mode of the control beam was by steel yielding, giving a large deflection of the beam providing sufficient ductility; however, for all repaired beams, the authors observed two failure modes: peeling off and interfacial debonding, which are a brittle and sudden failure. The authors concluded that all the repaired R.C. beams had a mechanical behavior in load capacity and rigidity higher than that of the control beam. In addition, it was determined that, for any damage degree, the CFRP laminates provide the repaired R.C. beam higher mechanical performances. The load capacity increases by 87% and 44% for damage degree 0% and 100%, respectively. The beam RB4 was completely damaged (pre-cracked to failure and large deflection: 10 mm), the contribution of CFRP laminate on the load capacity was very significant (144%). Furthermore, 80% and the 0% damage degree beams behaved likely, and they gave a higher performance in terms of load capacity and rigidity due to the additional contribution of the reinforced concrete. Finally, there was no rupture of the CFRP laminates for all repaired beams due to the lack of end anchorage.

[1] assessed the effectiveness of CFRP repaired R.C. beams under different damage levels. Experimental work is conducted on scaled beams where four beams were used as the datum. The first beam was without CFRP sheets (D.B.), the second was a repaired beam after pre-damaged under design load limit (RBD), while the third was a repaired beam after pre-damaged under steel yield load limit (RBY), and the fourth was a repaired beam after pre-damaged under ultimate load (RBU). The study used the flexural stiffness change based on the secant modulus of the load against deflection curves for comparison. Comparisons were made based on the flexural stiffness recovery, crack patterns, load capacity, and failure modes of the beams. The results showed that regardless of the pre-repair damage level, repairing with CFRP will be effective and increase the ultimate capacity.

Furthermore, results showed that enhancing the flexural stiffness and load capacity would be smaller with the increase in the pre-repair damage level. The CFRP repair technique increases the load capacity regardless of the pre-repair damage level, where it increases the load capacity by 83%, 56%, and 48% for the pre-repair damage levels of 35%, 66%, and 100%, respectively. It has been noted that the CFRP repair technique recovers the stiffness and increases it further than the undamaged stiffness, whereby it increases the stiffness by 17%, 10%, and 4.6% for the pre-repair damage levels of 35%, 66%, and 100% respectively. Finally, they concluded that failure modes are governed by the pre-repair flexural cracks, which induce an intermediate crack at the adhesive layer, thus causing CFRP debonding.

[3] investigated the load capacity, ductility, and energy absorption aspects of R.C. beams retrofitted after damage with sprayed-fiber reinforced polymer composites (SFRP). SFRP consists of randomly oriented chopped fibers of controlled length in a polymer matrix. The results indicated that SFRP could substantially increase strength and ductility and effectively strengthen and repair R.C. beams. Besides, it was concluded that carbon fibers lead to a higher increase in load-carrying ability and a lower increase in energy absorption for both damaged and undamaged R.C. beams due to their brittle characteristics compared to E-glass fibers.

[16] established a new green retrofitting material, called Green-USM-Reinforced Concrete (GUSMRC). The characteristics of this material (high tensile and flexural strengths) are very close to CFRP, which makes it suitable for retrofitting existing damaged concrete structures. Their study investigated the flexural behavior of damaged concrete beams caused by overloading when retrofitted by precast GUMSRC strips. The R.C. beams were damaged based on the ultimate load capacity of the control beams (25%, 50%, and 75%) and then rehabilitated. It was concluded that all retrofitting configurations significantly improved the strength of the beams in terms of a considerable increase in failure load.

By contrast, the mid-span deflection of retrofitted beams significantly decreased compared to the control beams, which had the highest deflections. Moreover, the effects of retrofitting at different damage levels were nearly the same.

[17] carried out an experimental investigation on the effect of using embedded CFRP rod as Near-surface Mounted (NSM) reinforcement for strengthening and repairing R.C. beams damaged by loading to different loading levels (0%, 50%, 70%, and 100% of ultimate load) and the results were compared with non-preloaded beams. Based on their findings, all the pre-loaded beams have lower stiffness than the control beams without pre-loading. However, the results indicated that the FRP reinforcement delays the crack propagation in the beams. The pre-loading levels have a minor effect on R.C. beams’ enhancement capacity, as beams pre-loaded to 50% and 70% show a decrease in enhancement level by 4.2% compared with strengthened beam without pre-loading, which could be neglected.

[18] developed a two-dimensional plane stress model of beams on the basis of experimental observation to investigate R.C. beams strengthened with pre-tensioned CFRP laminates under self-weight only and self-weight and external pre-load. It was concluded that the application of prestressed CFRP laminates is an efficient technique of strengthening R.C. flexural members, irrespective of the pre-loading level before strengthening. Although the pre-loading levels in two experimentally examined beams exceeded the serviceability limit states prior to strengthening, the application of prestressed CFRP laminates resulted in a significant reduction of deflections and strains due to subsequently applied loads. The prestressing technique led to partial recovery of beam stiffness similar to specimens without preloading. Also, the author conducted parametric studies. The simulations showed that preloading has a moderate effect on the load-bearing capacity. It was also demonstrated that, for elements made of high-strength concrete and with high pretension levels, the CRFP rupture failure mode may occur. The full strength of laminate can be utilized for such elements. The CFRP laminate width has an important influence on load-bearing capacity due to a greater prestressing force applied to the concrete section and a greater tensile force reached at failure. It is also worth noting that the laminate-rupture failure mode was obtained for the widest analyzed CFRP tape. The parametric study confirms the experimental observation concerning the strengthening efficiency of structures with different reinforcing steel ratios. It can be observed that the lower the reinforcing steel ratio is, the higher the reached level of strengthening efficiency. This effect is also valid in the case of passively strengthened beams.

The behavior of CFRP strengthened R.C. beams, and CFRP debonding mechanism is still an outgoing topic of research. Most of the research deal with the strengthening of R.C. beams without pre-damage. Therefore, more research is needed to investigate the behavior of repaired R.C. beams that experienced pre-repair flexure cracks.

This research aims to examine the flexural behavior and structural performance of pre-damaged reinforced concrete beams due to overloading, rehabilitated with externally bonded CFRP. The effect of pre-repair flexural cracks is examined on the bond between concrete substrates and CFRP sheets. The failure mode, load-deflection response, CFRP strain, and ductility of the bare and CFRP strengthened beams are discussed.

## 2. Experimental work

Six reinforced concrete beams with the same cross-section and span are cast and tested under four-point bending. The R.C. specimens are pre-loaded to certain strain levels in the main tensile reinforcement bars and then strengthened by a CFRP sheet. The materials used to cast R.C. beams are sand, gravel, water, cement, and reinforcement bars. Moreover, CFRP and epoxy resin were used to strengthen the R.C. damage beam externally. The properties of the materials obtained from testing in the lab based on ASTM and from Manufacture suppliers are presented in the following sections.

### 2.1. Concrete mix proportion

River sand from Derbandi Ranya with 2.64 specific gravity and 2.91 fineness modulus was used for the trail mix. In addition, the percentage of absorption is 1.432%. River gravel obtained from Peramagrun is used for the mixed proportions. The specific gravity, dry rodded unit weight, percentage of absorption, and nominal maximum aggregate size are 2.63, 1647 kg/m^3^, 1.31%, and 12.5mm, respectively. Ordinary Portland Cement (OPC) obtained from the Tasluja factory is used. Normal tap water was used for the mix proportions and curing of the specimens. The prepared materials (cement, sand, gravel, admixture, and water) were used to obtain trial mixes. The trial mixes for normal strength concrete were executed according to [19] based on the targeted compressive strength and workability. The sequence of mixing the materials and mixing time are essential keys to obtain a homogenous mix. The mixing sequence was as follows: first, the coarse aggregate was added inside the rotary mix, then half of the required water was added; second, all the fine aggregate was added to the mixer, followed by the cement. Finally, the remaining water was added gradually until a homogenous mixture was obtained. For Normal Strength Concrete (NSC), the materials were mixed for 3 minutes, followed by 3 minutes rest, followed by 2 minutes mix again. Table 1 illustrates the selected mix proportions and weight per cubic meter of each material used for casting R.C. beams.

**Table 1:** Selected mix proportions for casting R.C. beams.

Mix Proportions	Types of Concrete	Mix Ratio C:S:G	W/C ratio	Cement (kg/m^3^)	Sand (kg/m^3^)	Gravel (kg/m^3^)	F/C*	fc’ (MPa)	Slump (mm)
**I**	NSC*	1:1.8:1.56	0.43	480	854	748.8	1.140	45	100
		Water =	(216kg/m^3^)						

*F/C: the ratio of fine to coarse aggregate.

*HRWR%: High Range Weight Reducer as a percentage of cement.

*NSC: Normal Strength Concrete.

### 2.2. Concrete test results

Table 2 illustrates the average value of the mechanical properties of the concrete specimens. A set of cubes, cylinders, prisms were tested according to ASTM standards and EN BS standards to acquire cylindrical and cubical compressive strength, splitting tensile strength, modulus of rupture, and modulus of elasticity. The cylinders were capped according to [20].

**Table 2:** Mechanical properties of the concrete beams.

Mechanical Properties	Results
	B1-B6
**f’c (MPa)**	43
**fcu (MPa)**	63
**f’c/fcu**	0.7
**fr (MPa)**	5.5
**fsp (MPa)**	3.9
**E (GPa)**	31

**Fig 1:**
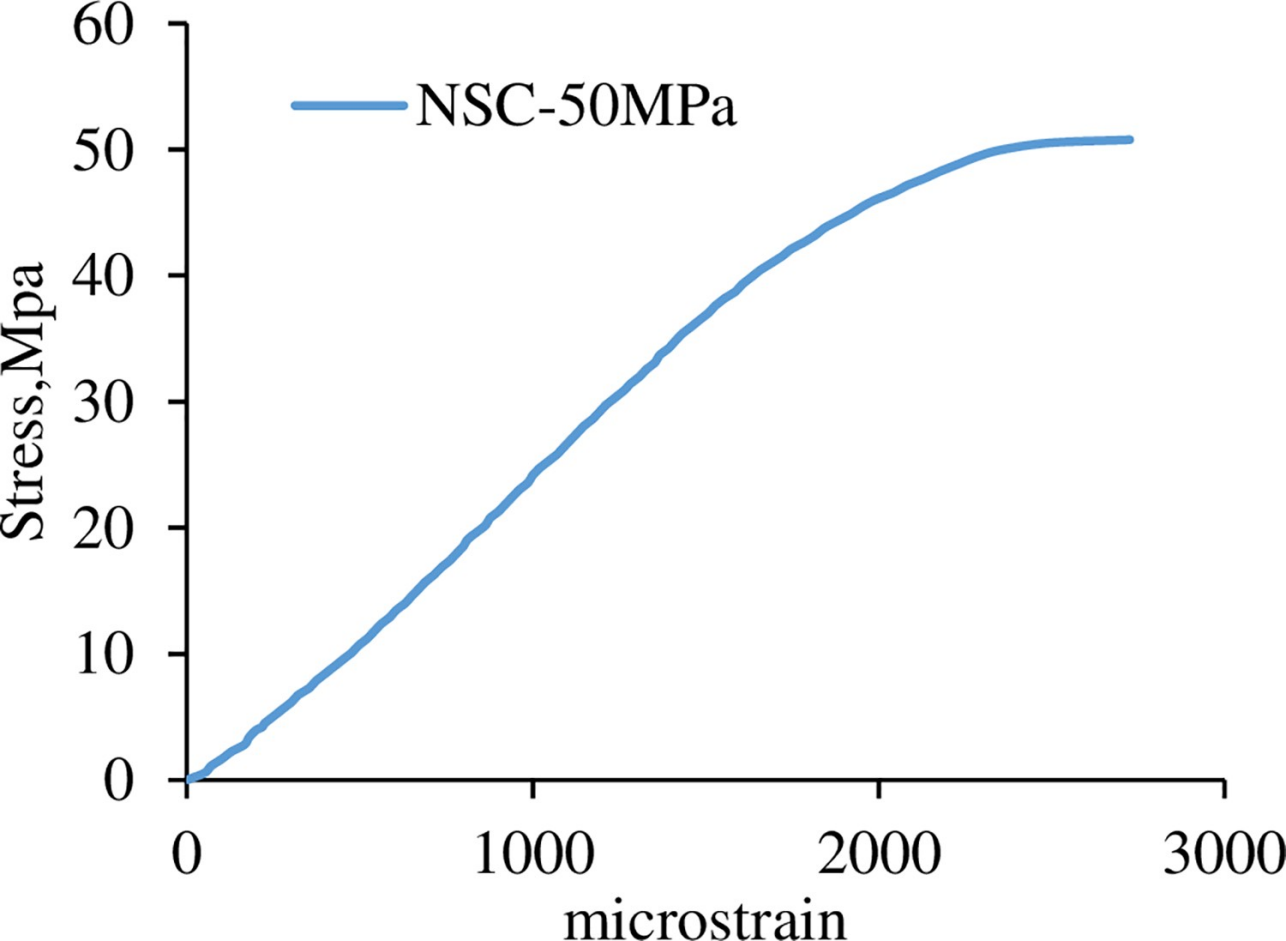
Stress-strain curve of NSC using strain gauges.

**Fig 2:**
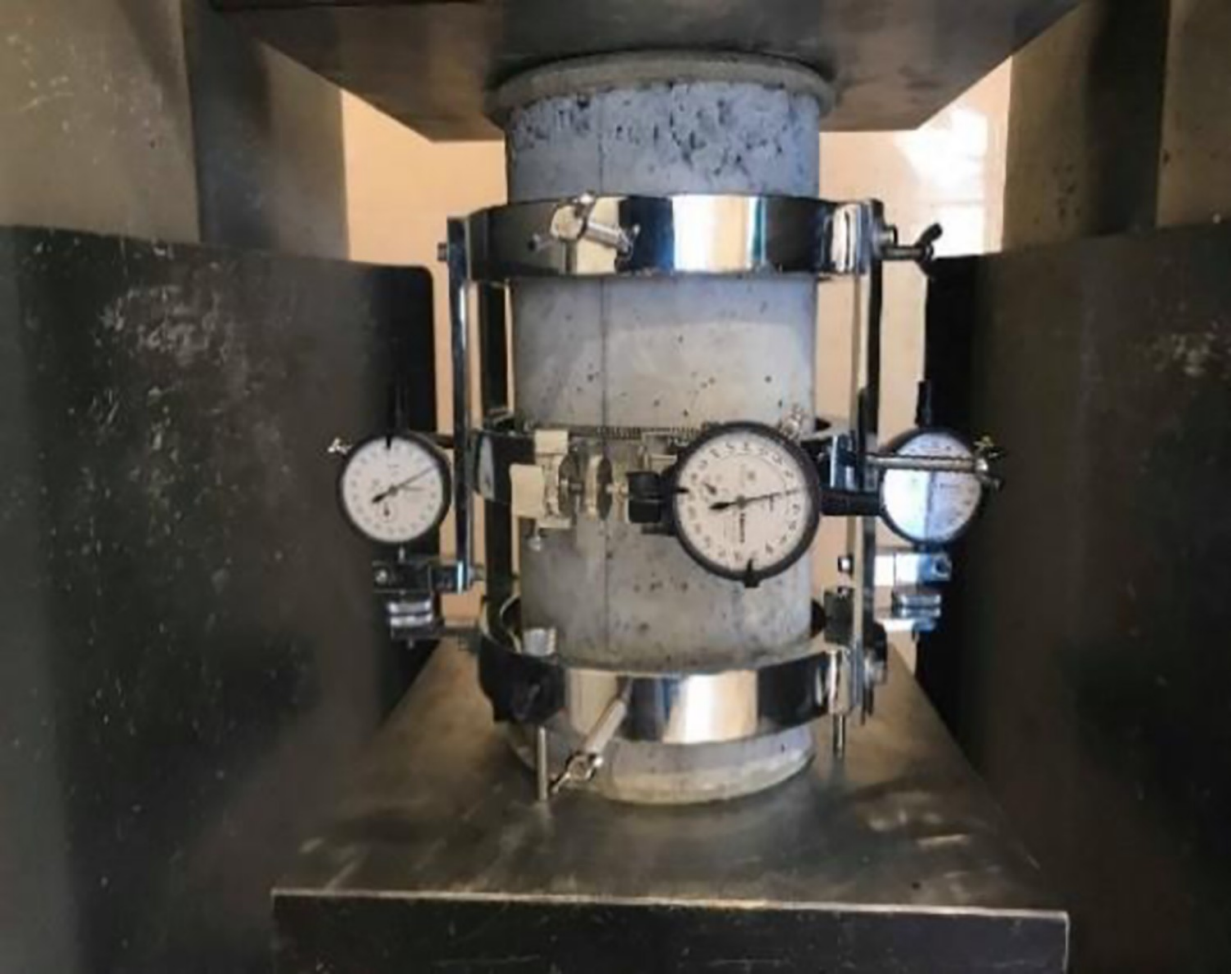
Modulus of elasticity test using compressometer.

Fig 1 shows the concrete’s stress-strain behavior obtained by installing two 80mm gauge length strain gauges on two opposite sides of the (150mm x 300mm) cylinder under compression. The concrete’s modulus of elasticity (E) was measured under compression, using compressometer according to [21], as shown in Fig 2.

### 2.3. Steel reinforcement

**Fig 3:**
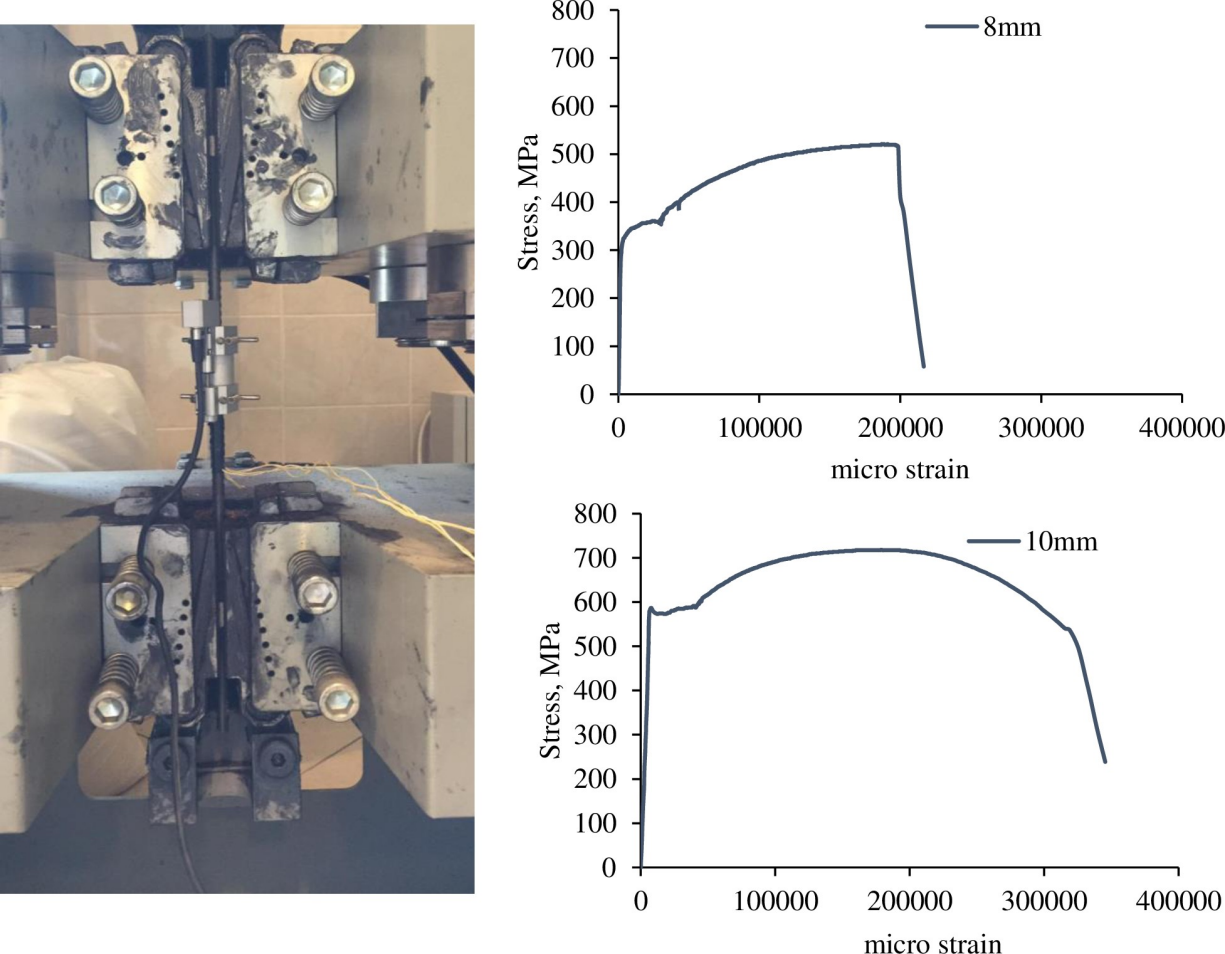
Tensile test of steel bars using extensometer and strain gauges and stress-strain curve for 8mm and 10mm bar.

Samples of deformed steel bars used for main tensile reinforcement and stirrups in the R.C. beams were prepared and tested according to [22]. The steel bars were obtained from the Mass factory. Extensometer was installed on the samples with the universal tensile machine to achieve the stress-strain curve of each bar and measure the modulus of elasticity, as shown in Fig 3. Moreover, two strain gauges were installed on the samples to verify the results from the extensometer. Table 3 illustrates the measured diameter and mechanical characteristics of 8mm and10mm bars.

**Table 3:** Mechanical properties of the reinforcement bars.

Reinforcement bar	8mm			10mm		
	Mean	Standard Deviation	COV	Mean	Standard Deviation	COV
**diameter (mm)**	7.68	0.057	0.0074	9.77	0.0147	0.0015
**fy (MPa)**	330	28.46	0.0862	573	11.49	0.0201
**fu (MPa)**	524	15.99	0.0305	711	8.43	0.0119
**E (GPa)**	165	13.79	0.0836	164	29	0.1768
**elongation %**	23	5.41	0.2234	20.6	7.54	0.3656

### 2.4. CFRP and adhesive

**Fig 4:**
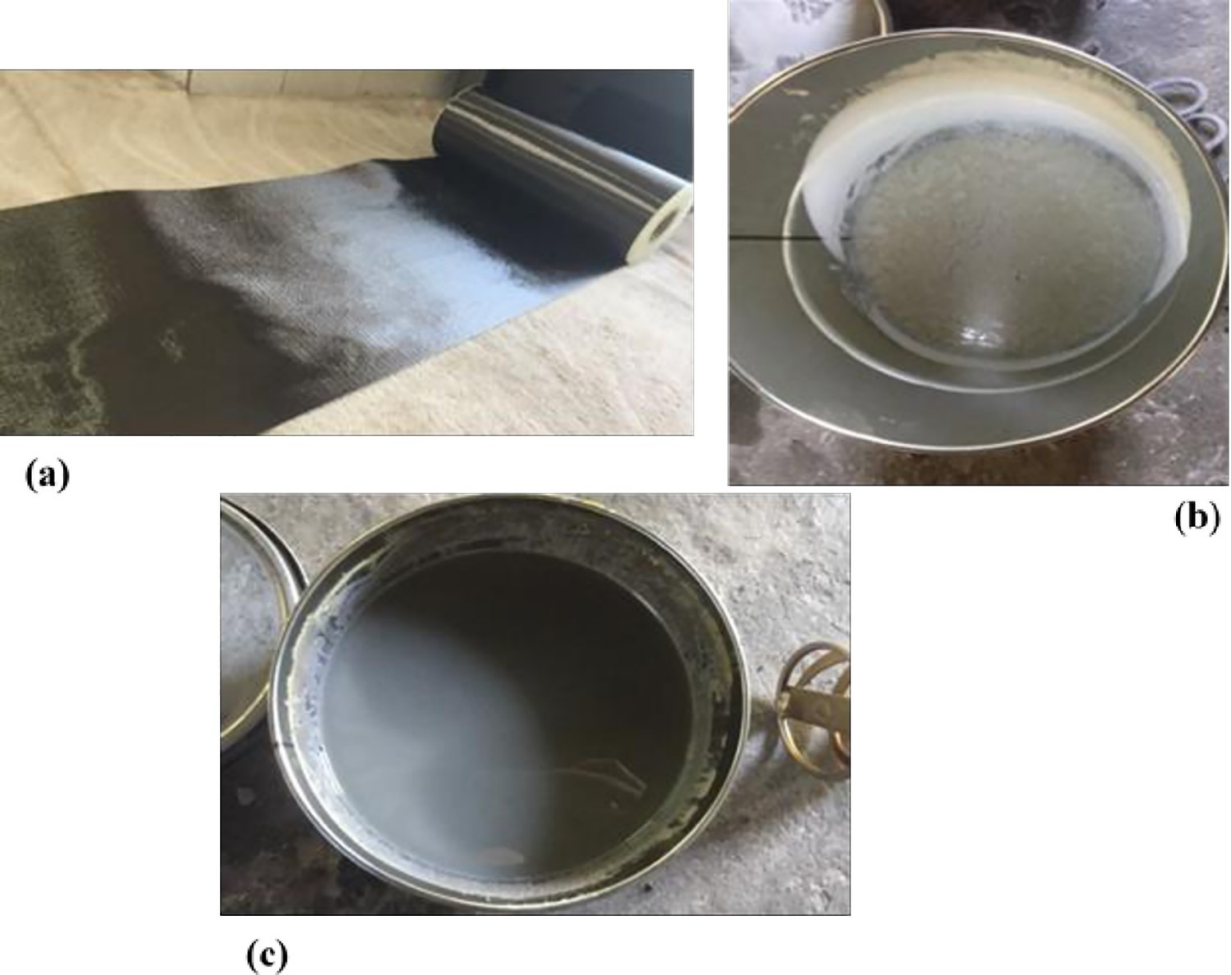
CFRP and epoxy resin: (a) CFRP roller; (b) epoxy resin component A; and (c) epoxy resin component B.

ASOFABRIC-C300 carbon fiber fabric with ASODUR-1330 two components (A and B) epoxy adhesive products of AB-SCHOMBURG was used to strengthen the pre-damage R.C. beams. The carbon fiber fabric has 50cm width and 100m length rolled, as shown in Fig 4. Table 4 illustrates the CFRP and epoxy adhesive properties based on the technical datasheet.

**Table 4:** Properties of CFRP and epoxy adhesive based on the technical datasheet.

CFRP (ASOFABRIC-C300)		Epoxy Adhesive (ASODUR-1330)	
Thickness (mm)	0.166	Basis	two-component epoxy resin
Ultimate tensile strength (MPa)	4900	Color	amber
E (GPa)	230	Mixing ratio	3:1 (by volume, A: B)
Elongation at break (%)	2.1	Tensile strength (MPa)	55
Areal Weight (g/m^2^)	300	Tensile Modulus (MPa)	1.7
		Elongation at break (%)	3
		Flexure strength (MPa)	79

### 2.5. Specimen’s design

**Fig 5:**
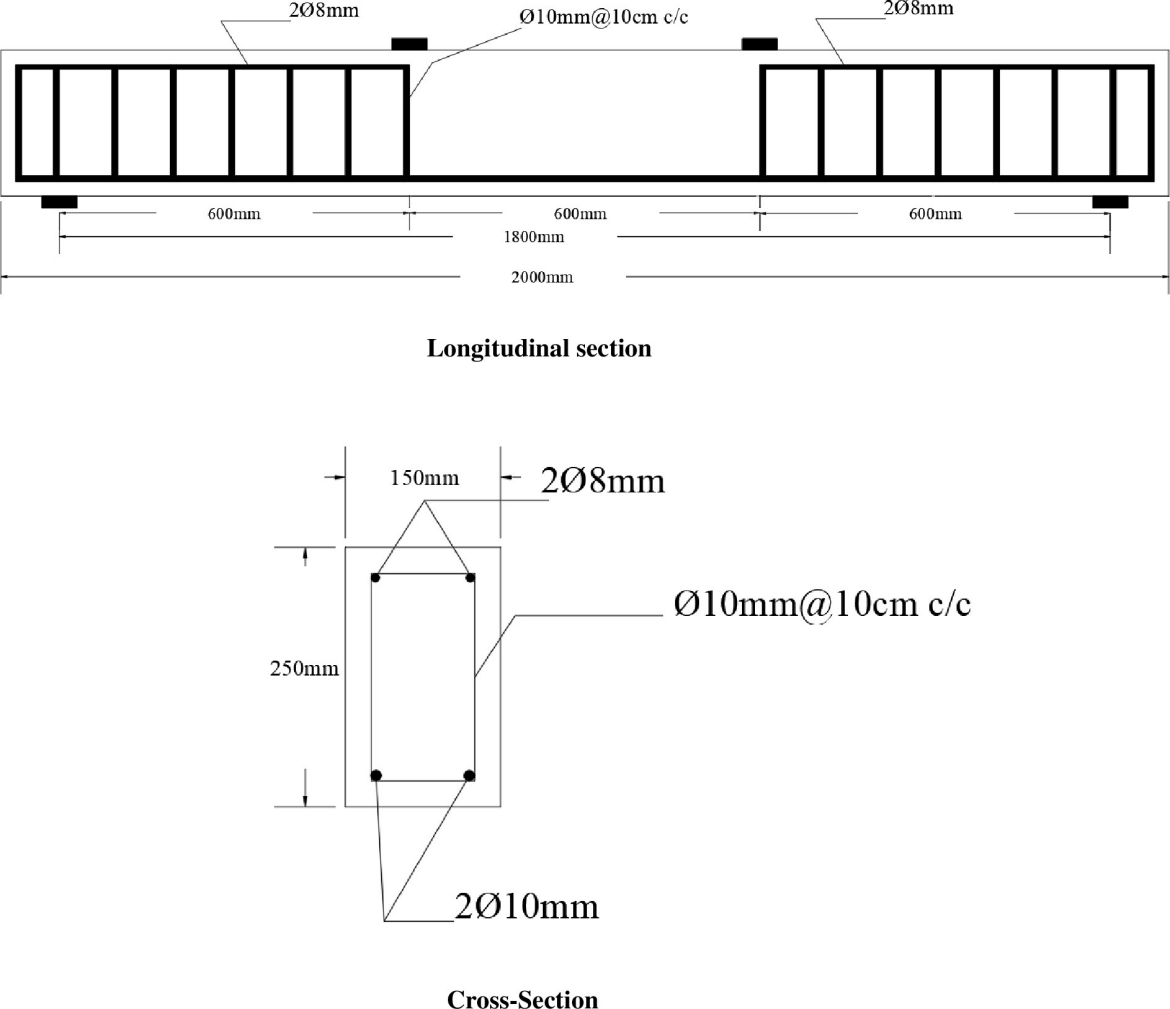
Longitudinal and cross-section of the R.C. beams.

All the R.C. beams have identical cross-sections and spans (150mm x 250mm x 1800mm). R.C. beams were designed according to [23], intentionally overdesigned in shear so that the specimens will fail in flexure. The study parameter is pre-damage level, as presented in Table 5 with the corresponded beam labels. Fig 5 shows the longitudinal section and cross-sections of the R.C. beams. The top bars were discontinued to the constant moment region so that the compression force will be resisted by the concrete only. The concrete surface and the CFRP sheet have the same width, equal to (150mm).

**Table 5:** Details of the R.C. beams.

RC Beams	Damage Level up to strain in the main bars
**B1 (Control)**	
**B2**	0
**B3**	1800microstrain
**B4**	2400microstrain (Yield)
**B5**	3000microstrain
**B6**	4000microstrain

### 2.6. Casting R.C. beams

Six plywood molds and reinforcement cages were constructed and prepared for casting the R.C. beams, and 25mm plastic covers were used for the bottom and side cover of the reinforcement bars. The plywood molds were oiled before concrete casting to protect the molds and remove the molds easily after concrete hardening. A laboratory rotary mixer was used for mixing the materials. An electrical vibrator was used to vibrate and compact the fresh concrete in the molds to minimize air voids and segregations.

After the concrete was cast, the top surface of the fresh concrete was leveled and smoothed. The R.C. beams were left for 24 hours in the molds covered with wet gunny sacks. The hardened R.C. beams were removed from the molds and water cured for seven days and left for 28 days before the pre-loading process to gain full concrete strength. The R.C. beams were painted using white color water emulsion painting and labeled using the marker. The reason for the painting is to notice the crack when it occurred.

### 2.7. Pre-loading

**Fig 6:**
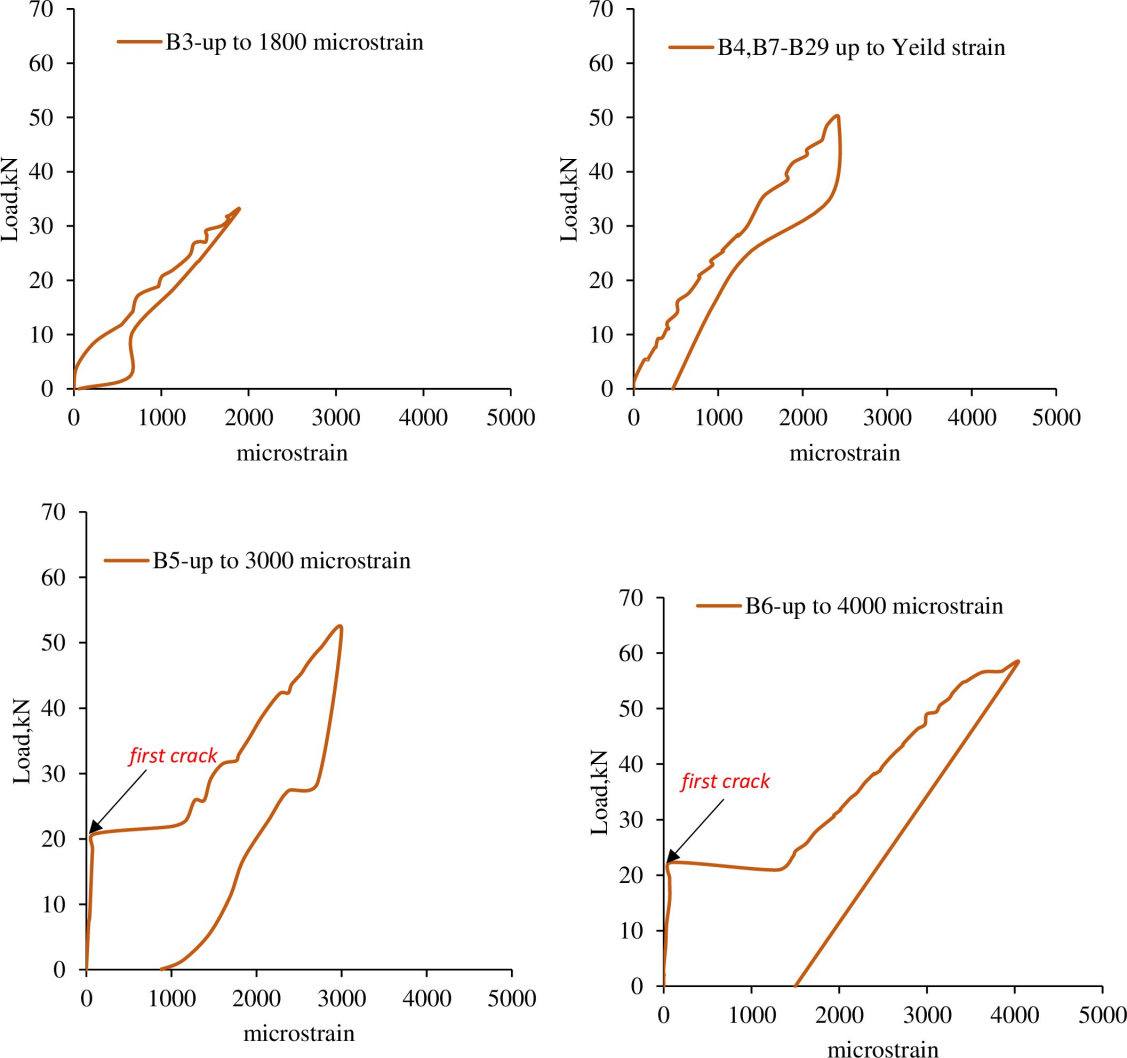
Load-strain relation of the damaged R.C. beams.

All the R.C. beams except for B1 and B2 were loaded and damaged before strengthening. The damage levels were based on the strain in the main tensile bars of the R.C. beams. Four different damage levels were conducted depending on the specific strain in the tensile bars. B3 was damaged up to 1800microstrain; however, B4 was damaged up to yield strain (2400microstrain) of the main bar. Moreover, B5 was loaded up to 3000microstrain, and B6 was loaded up to 4000microstrain. Fig 6 shows loading and unloading versus strain of the R.C. beams before strengthening.

**Fig 7:**
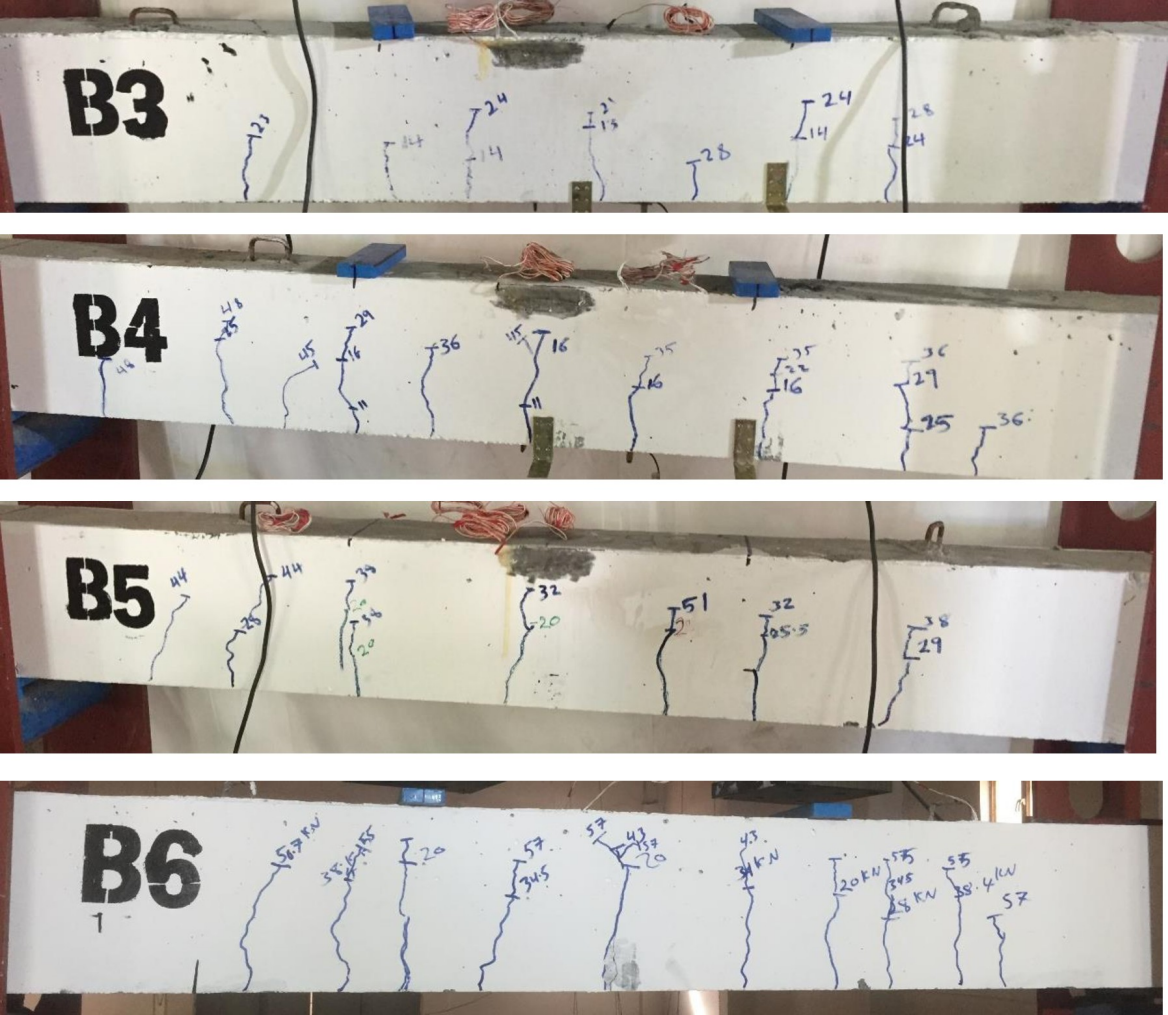
Crack pattern of R.C. beams after damage for different damage levels.

Fig 7 shows crack patterns for R.C. beams with different damage levels. The cracks are flexural as expected because the R.C. beams are over-designed in shear. The first crack occurred under one of the point loads. For B3 and B4, the first crack appeared around 14kN and 16kN, respectively. Furthermore, as is evident in the load-strain relation for B5 and B6, the first crack occurred at a load of 21kN.

### 2.8. CFRP strengthening

Before applying CFRP and epoxy resin on the concrete tension face, dust, piece of extra concrete, water emulsion paint, and moisture should be removed using a mechanical grinder and brush. Therefore, the R.C. beams were overturned on the bottom surface. The surface was washed using water and dried using an air blower before CFRP application.

The pre- damaged R.C. beams were strengthened with 150mm width of one layer of CFRP in the tension face. The two-component resin A and B were mixed and applied on concrete surfaces and CFRP sheets, and then the saturated CFRP sheet was applied on the concrete surface. A wooden roller was slowly used to press out air and extra resin. Based on the manufacturer’s technical report, the strengthened beams were left for 28 days in the laboratory to harden the epoxy resin.

### 2.9. Instrumentation

**Fig 8:**
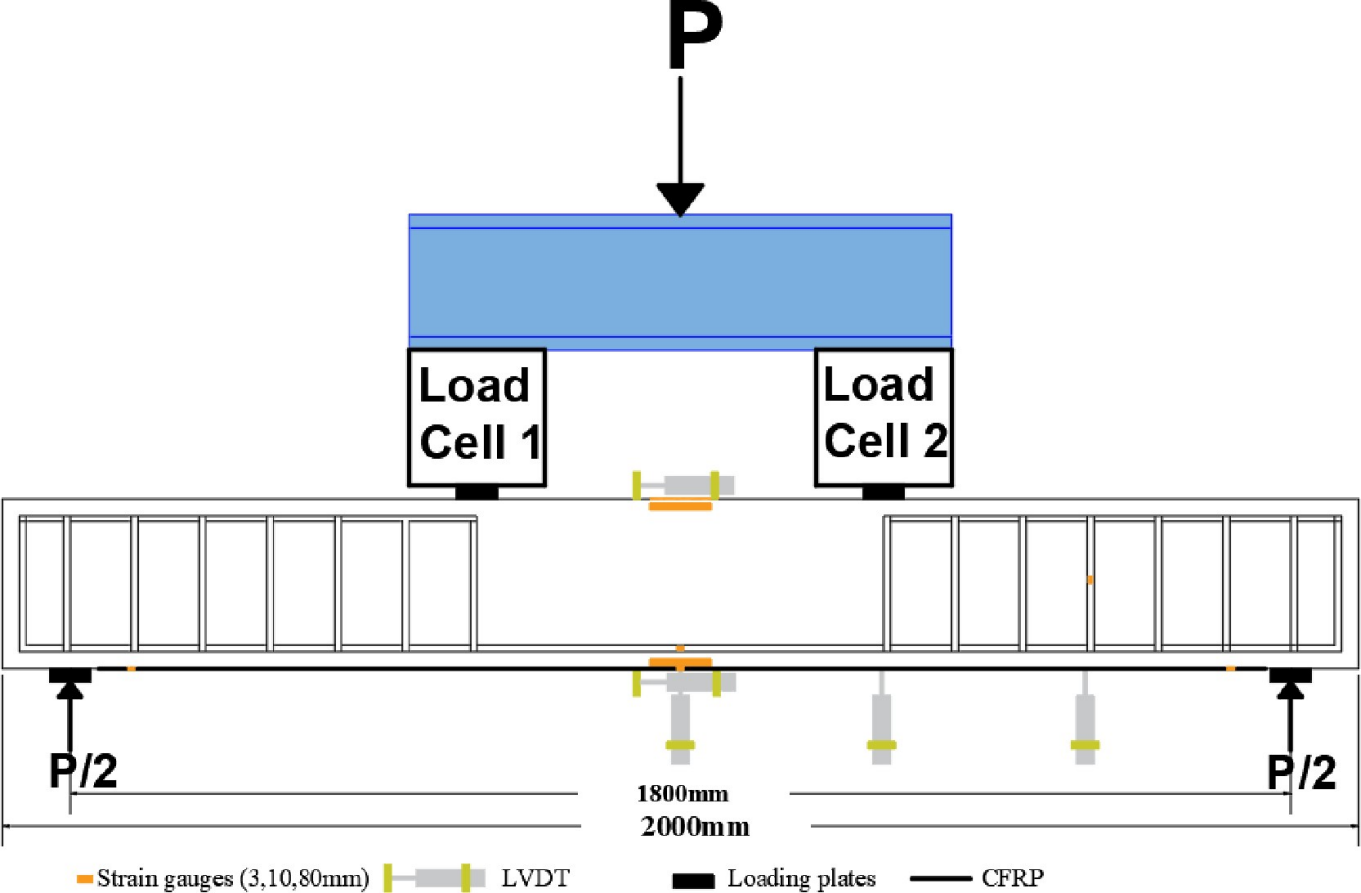
Loading setup and instruments.

Fig 8 shows a schematic representation of the loading setup and instrumentation. The strengthened R.C. beams were tested under four-point bending using a hydraulic jack of 800kN capacity. The pressure from the jack is divided into two-point loads using an I-section steel beam with two load cells of 300kN capacity each. Four steel plates (60mm x 200mm x 20mm) were used under the load cells and on the supports to prevent local failure due to stress concentration.

Three different gauge lengths of strain gauges were installed, 3mm strain gauges installed on the main tensile bars at mid-span and on the fourth stirrup from the support to read the tensile strain of the bars. 10mm strain gauges were installed on the CFRP sheets at mid-span and near the supports to measure the tensile strain of the CFRP sheet. Furthermore, 80mm strain gauges were installed on the top, top face, and bottom of the concrete section at mid-span to measure the concrete’s top compressive strain and bottom tensile strain.

Five Linear Variable Differential Transformers (LVDT) were used to measure the deflection at mid-span, under the point load, and mid-way between the point load and the right support and to measure top concrete compressive strain and bottom tensile strain.

Windmill MicroLink, 851 data logger with 594 box (screw terminals with strain measurement), were used to collect and read the load cells, LVDTs, and strain gauges.

## 3. Experimental results and discussion

Five R.C. beams (B2-B6) with different pre-loading levels were tested to investigate the effect of different pre-damage levels on the failure mode, crack pattern, load-deflection curve, strain contribution of CFRP, and ductility of the CFRP strengthened R.C. beams. The specimen B2 is strengthened without pre-loading; B3, B4, B5, and B6 were pre-loaded up to 53.7%, 81.2%, 84.5%, and 97.4% of the ultimate load capacity of the control beam before FRP strengthening.

**Fig 9:**
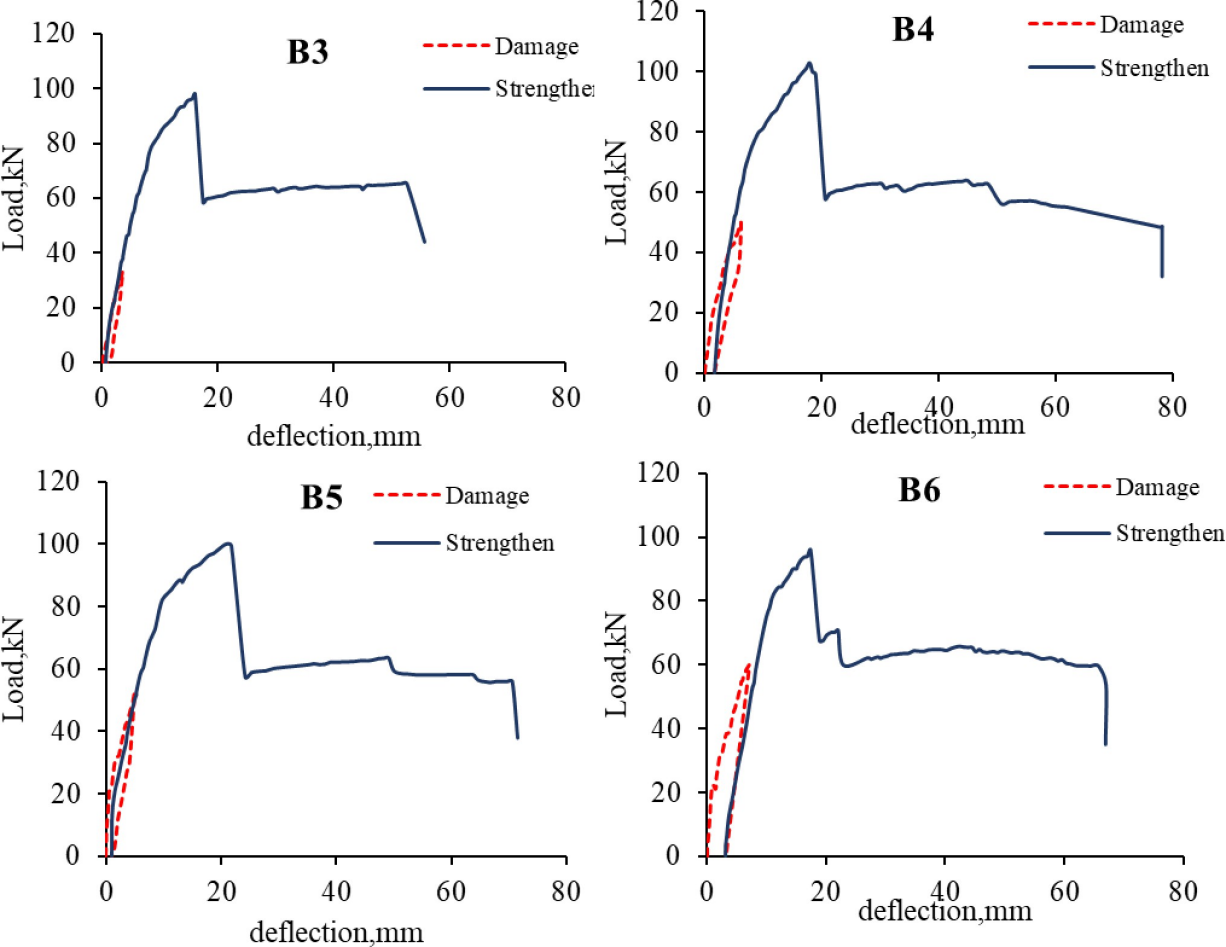
Load-deflection curve for pre-damage and strengthened beams.

Fig 9 shows the loading and unloading process and the load-deflection curve of the strengthened R.C. beams after damage. The test results of the strengthened R.C. beams are compared with the control beam B1.

### 3.1. Mode of failure and crack patterns

**Fig 10:**
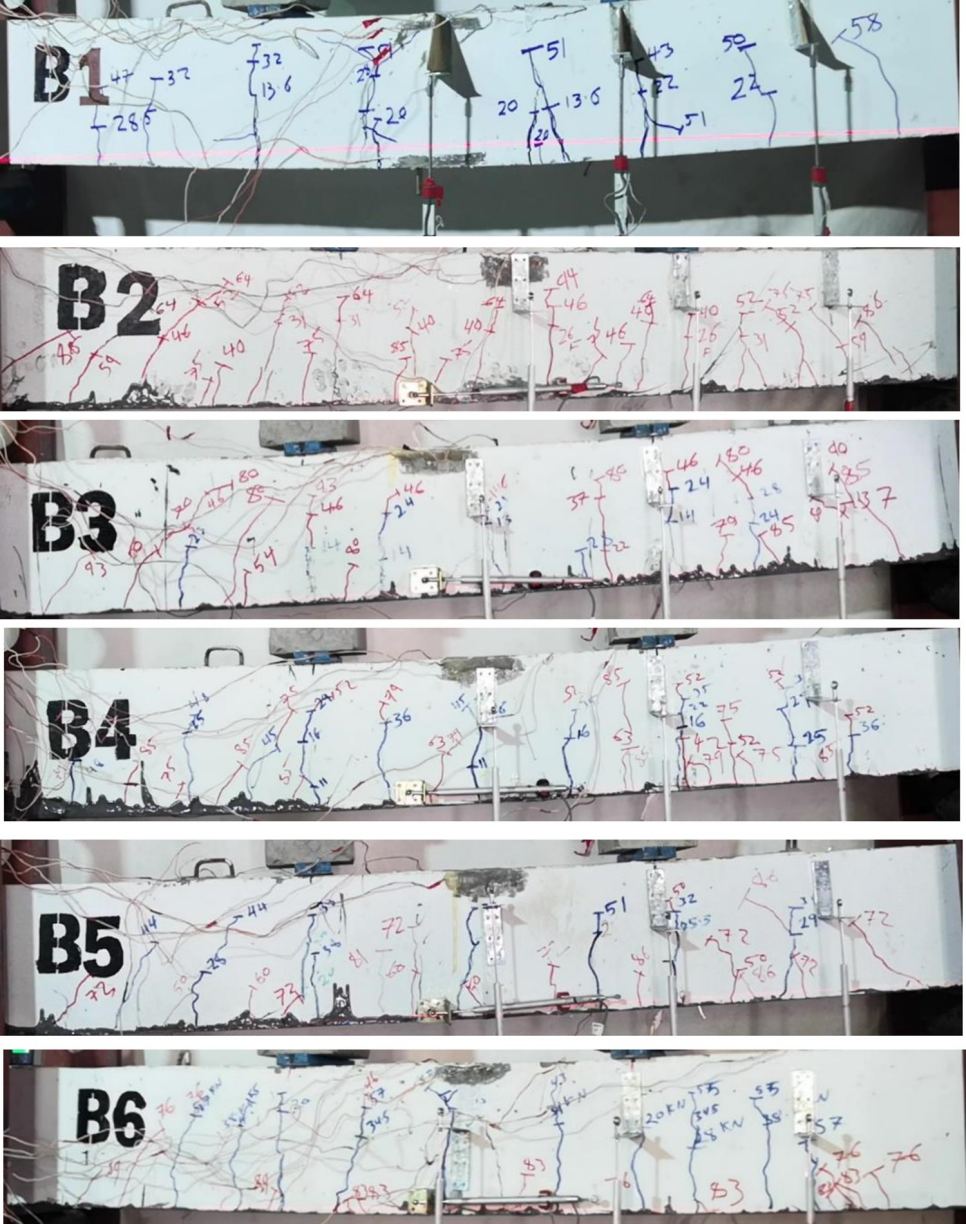
Crack patterns before failure.

**Fig 11:**
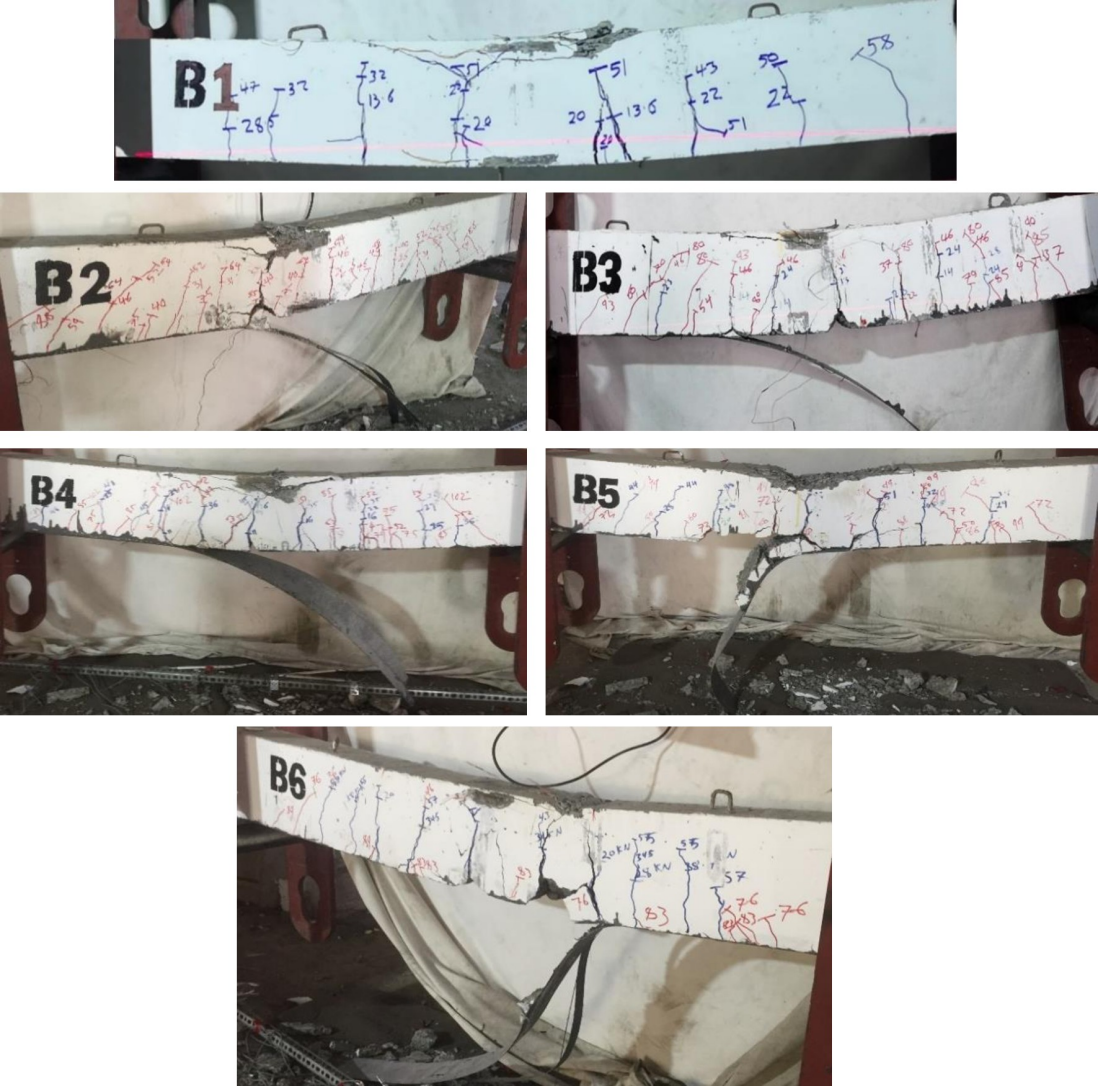
Failure mode of the R.C. beams: Concrete crushing, B1; FRP end peeling off, B2-B4; and concrete cover separation, B5 and B6.

The control beam experienced a flexure crack mostly under and between the point loads (constant moment region) and propagated towards the support, as shown in Fig 10. With increasing the applied load, top concrete fibers reached the ultimate strain, and the control beam failed in concrete crushing after yielding the steel bars, as shown in Fig 11, which is a typical tension control failure according to ACI-318. Before the failure, B1 underwent deflection and had ductile behavior.

Furthermore, the strengthened R.C. beams (B2-B6) suffered from more flexure cracks than B1 before failure; as shown in Fig 10, the width of the old cracks was increased, and the new cracks appeared. The cracks initiated under the point load and distributed along the R.C. beams. Moreover, the cracks propagate towards the compression region, especially in beams B5 and B6; more cracks reached the compression region. At the final stage of loading before failure, some diagonal cracks, indicating shear cracks, near the supports initiated in the strengthened R.C. beams due to the increase in the flexure capacity of CFRP reinforced beams.

All the strengthened R.C. failed in CFRP debonding; CFRP debonding was initiated near the supports and propagated toward mid-span. Before CFRP debonding, the epoxy resin fractured because of reaching its tensile capacity.

**Fig 12:**
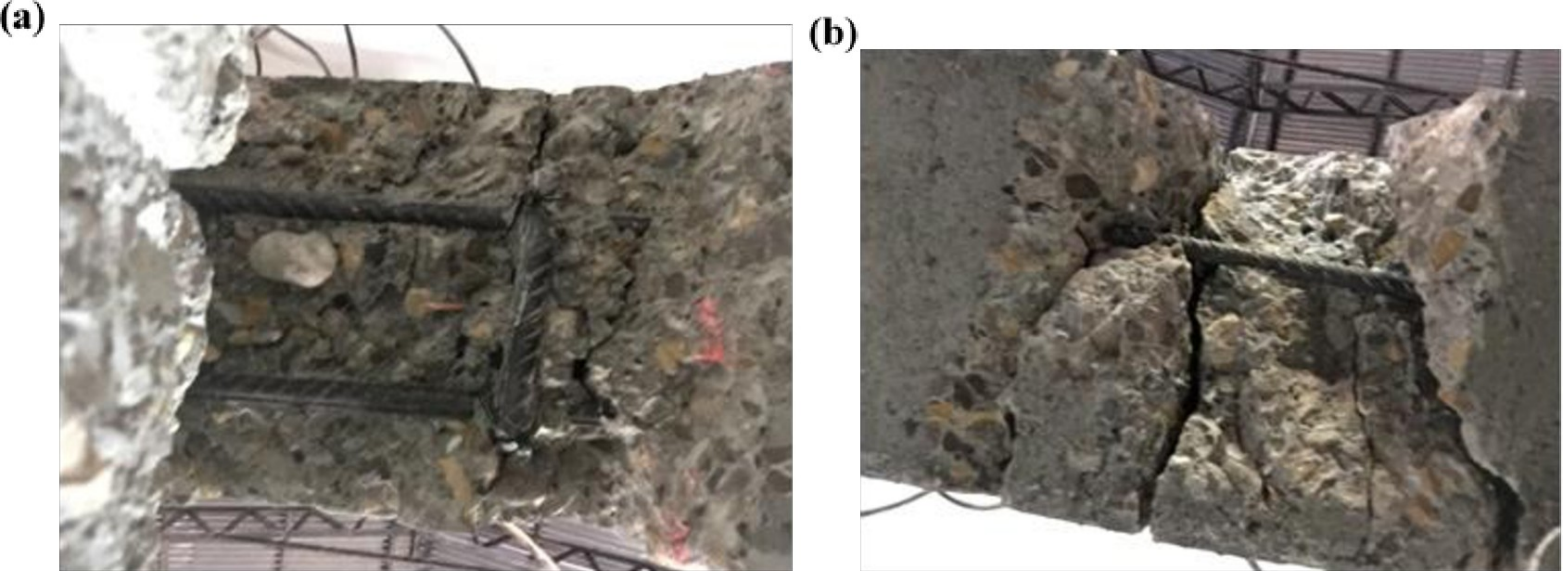
Cover separation bottom view; (a) B5, and (b) B6.

The beams B2-B4 failed in CFRP end peeling off, as shown in Fig 11, without cover delamination. However, specimens B5 and B6 failed in concrete cover delamination, as shown in Fig 11; this may be because the concrete cover was weakened because the concrete surface was more damaged and experienced wide cracks during the pre-loading process before applying CFRP. Cover delamination occurred under one of the point loads in which a part of concrete was separated from the R.C. beams attached to the FRP, and the reinforced bar appeared, as shown in Fig 12. After FRP debonding, loading was continued until concrete crushing occurred.

### 3.2. Load-deflection curve

Table 6 presents the load and mid-span deflection of specimen B1-B6 at critical loading stages. The first crack of specimen B1 occurred at a load of 14.3kN with a corresponded mid-span deflection of 1.79mm. However, in specimen B2, the first crack appeared at a higher load of 26.8kN with a lower corresponded mid-span deflection of 1.36mm. Thus, it can be concluded that CFRP delays the first crack and increases the cracking load by 87.4%. Furthermore, in the strengthened R.C. beams, the steel bar yields at a higher load than B1; for instance, in B2, the steel yielding load increased by 34.4%.

**Table 6:** Mode of failure and load and mid-span deflection at first crack, the yield of steel, and the ultimate stage.

R.C. beams	first crack		yield of steel		ultimate		Mode of Failure
	Load (kN)	δ (mm)	Load (kN)	δ (mm)	Load (kN)	δ (mm)	
**B1**	14.3	1.79	55.0	12.81	61.6	47.20	C.C
**B2**	26.8	1.36	73.9	7.85	101.0	17.73	F.D+C.C
**B3**			68.1	6.50	98.1	15.36	F.D+C.C
**B4**			80.0	7.62	102.9	17.31	F.D+C.C
**B5**			72.2	7.41	100.0	20.75	F.D+C.C
**B6**			76.9	7.17	96.0	14.27	F.D+C.C

CC: concrete crushing, F.D.: CFRP debonding

**Fig 13:**
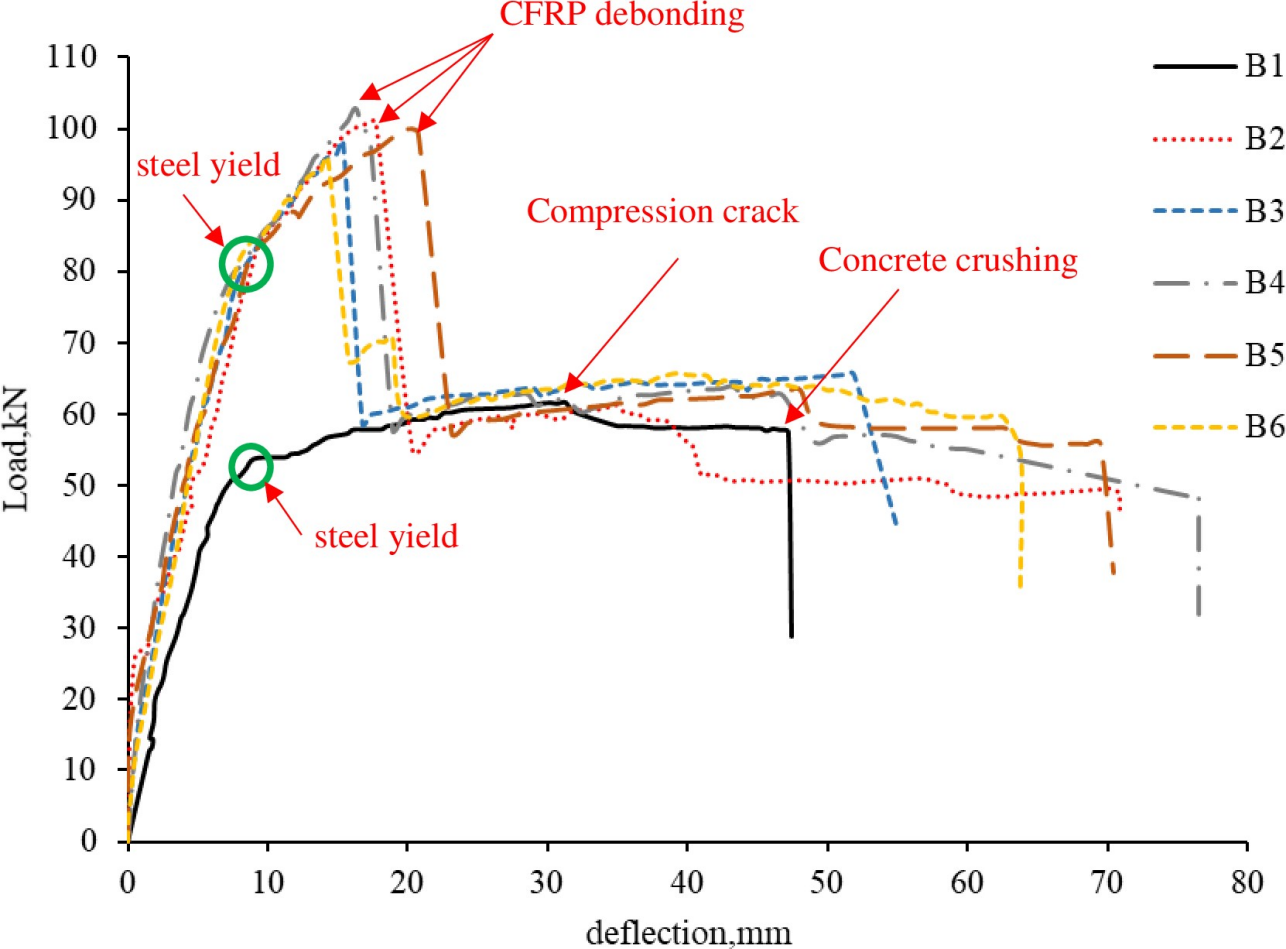
Load-deflection curve.

**Fig 14:**
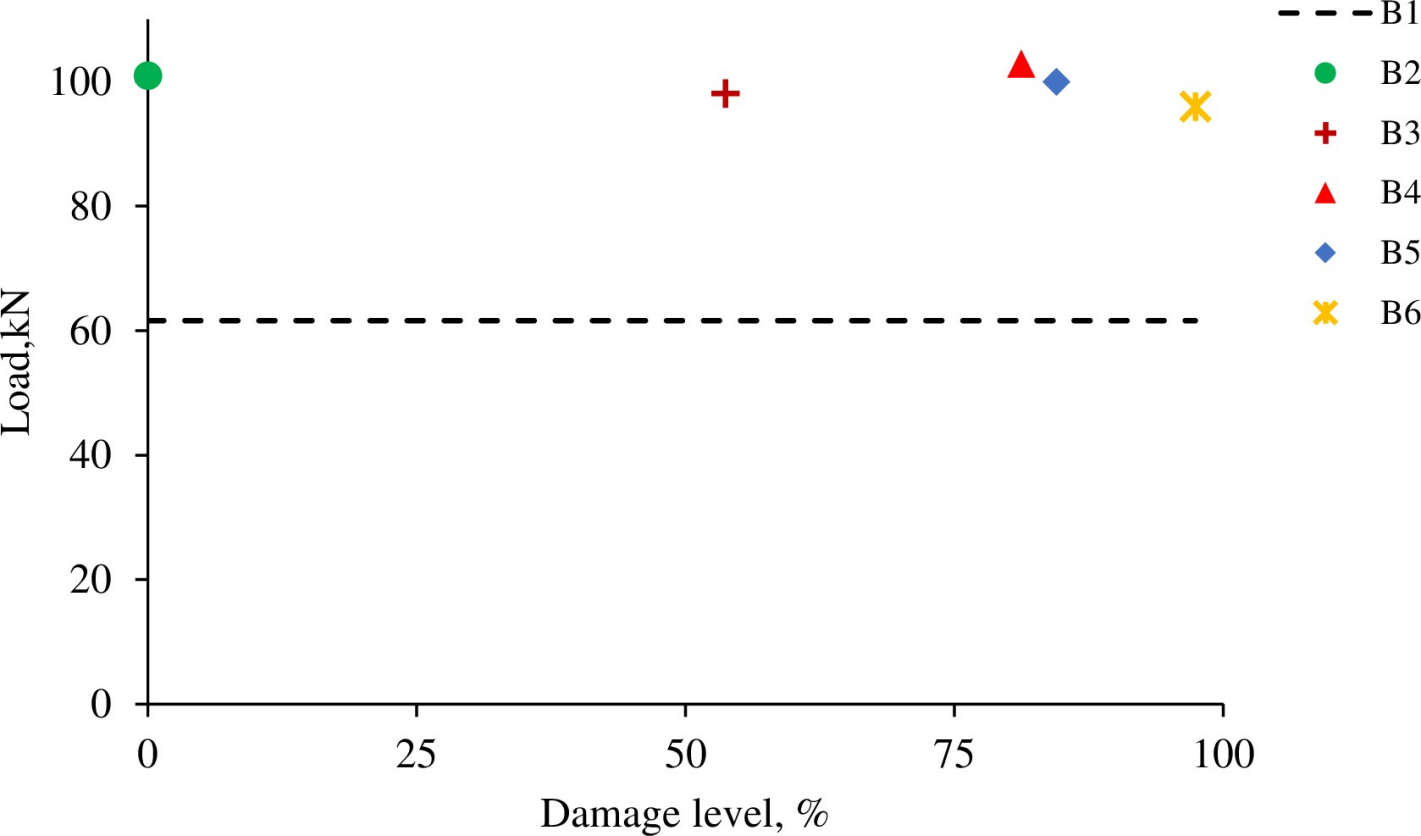
Ultimate load and damage levels.

All the strengthened R.C. beams, B2-B6, have higher ultimate load capacity and lower mid-span deflection than specimen B1, as shown in Figs 13 and 14. For beams B2-B6, the maximum load capacity was increased by 64%, 59%, 67%, 62%, and 56%, respectively. However, the mid-span deflection at failure for beams B2-B6 was decreased by 62%, 67%, 63%, 56%, 70%, respectively. Specimen B4 has the highest ultimate load among all the strengthened R.C. beams, and specimen B6 has the lowest capacity. It can be concluded that the increase of the ultimate load capacity of the CFRP strengthened beams is independent of the pre-loading level.

**Fig 15:**
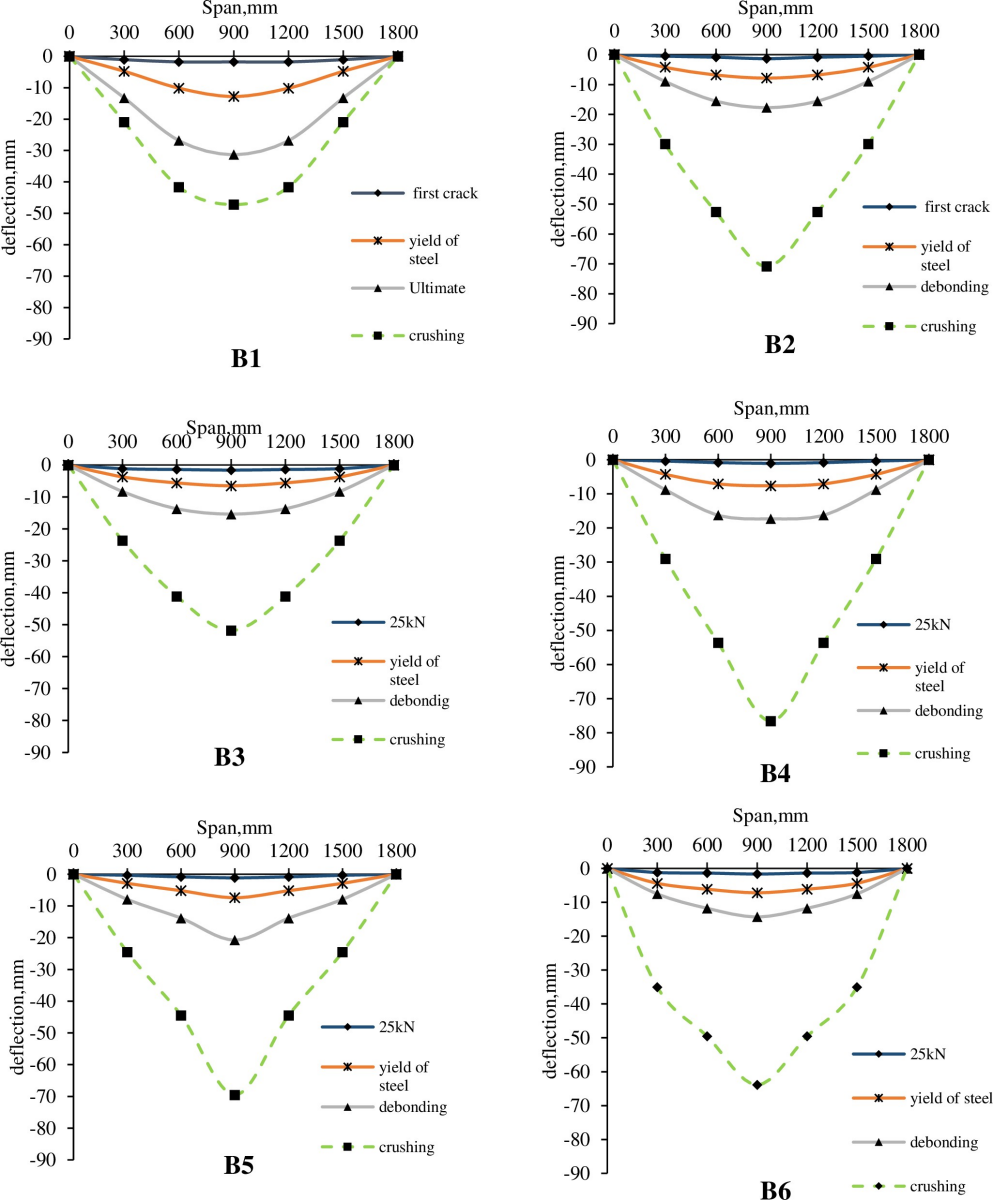
Deflection curve at critical loads.

The post debonding behavior of the strengthened R.C. beams was similar to the behavior of the control beam. Fig 15 shows the mid-span deflection curves of specimen B1-B6 at different load levels. Fig 15 was produced by assuming the remaining half of the span to deflect symmetrically by the same amount as the instrumented one. The mid-span deflection of B2-B6 was lower than B1 at first crack load, the yield of steel load, and ultimate load since CFRP increases the flexure rigidity of the R.C. beams. However, after CFRP debonding, the strengthened R.C. beams have more deflection up to concrete crushing than the control beam because the strengthened R.C. beams experienced more and wider cracks before debonding, and the R.C. beams lost most of the flexure rigidity after CFRP debonding.

### 3.3. Strain characteristics

**Fig 16:**
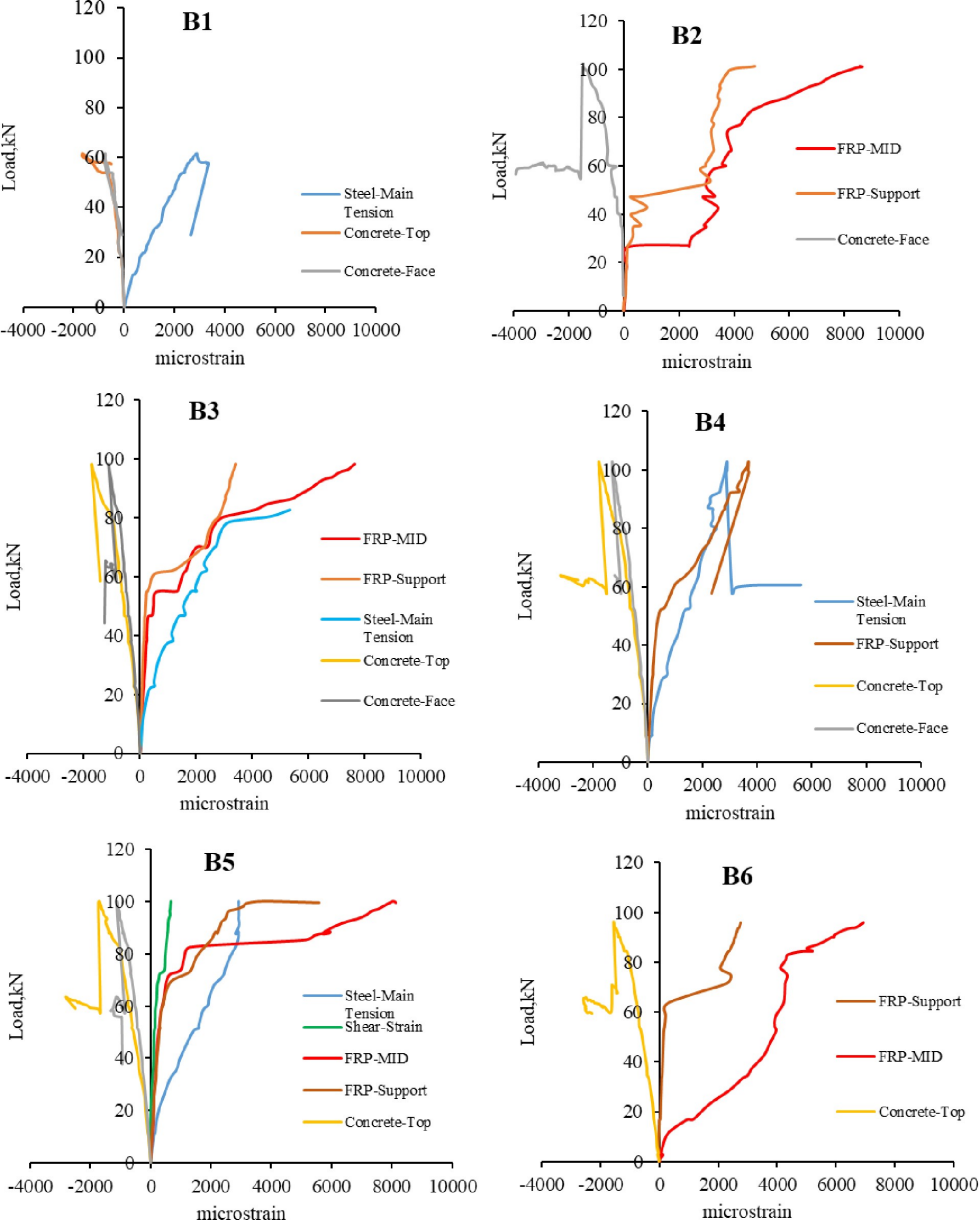
Load-strain curve.

Table 7 presents the strain in the top concrete fiber, steel bars, and CFRP sheet at mid-span at failure. Fig 16 illustrates the load-strain curve of concrete, tensile steel bar, and CFRP sheet.

**Table 7:** Strain of concrete, steel, CFRP at failure.

R.C. beams	ε_c_ (microstrain)	ε_s_ (microstrain)	ε_f_ (microstrain)	ε_fu_ (microstrain)	ε_f_/ε_fu_%
**B1**	-1665	3334			
**B2**	-----	-----	8642	21000	41.2%
**B3**	-1723	5342	7646	21000	36.4%
**B4**	-1783	2885	8025	21000	38.2%
**B5**	-1701	2931	8134	21000	38.7%
**B6**	-1553	-----	6939	21000	33.0%

ε_c_: concrete strain, ε_s_: steel strain, ε_f_: CFRP strain, and ε_fu_: CFRP rupture strain.

The yielding strain of the steel bar is 2400 microstrain, so the steel bars yielded before failure in all the R.C. beams. The strain result is not presented because some of the strain gauges were damaged or did not read the value properly. Since the concrete crushing for B1 occurred near the right point load, the top concrete strain gauge did not reach the strain failure of concrete at mid-span. Similarly, for B3, the steel strain gauge provided a higher value because the wider flexure crack occurred at mid-span.

The rupture tensile strain of CFRP (ε_fu_) provided by the manufacture datasheet is 21000 microstrain. In all the CFRP strengthened beams, CFRP strain at debonding was between 41.2%-33% of the FRP rupture strain. B2 gives the highest CFRP strain before debonding; however, B6 gives the lowest CFRP strain. The strain contribution of CFRP decreases with increasing the damage level because the concrete substrate is weakened, and the pre-repair flexural cracks induce intermediate cracks at the adhesive layer and cause early debonding before reaching the CFRP sheet to its yielding strain. For B2 with zero damage level, the CFRP strain reached 41.2% of the CFRP rupture strain. The ratio of CFRP strain to the strain of rupture for B3, B4, and B5 were approximately the same 36.4%, 38.2%, and 38.7%, respectively. Beam B6, with a higher damage level, gives the lowest ratio, 33%.

### 3.4. Ductility, toughness, and stiffness

**Fig 17:**
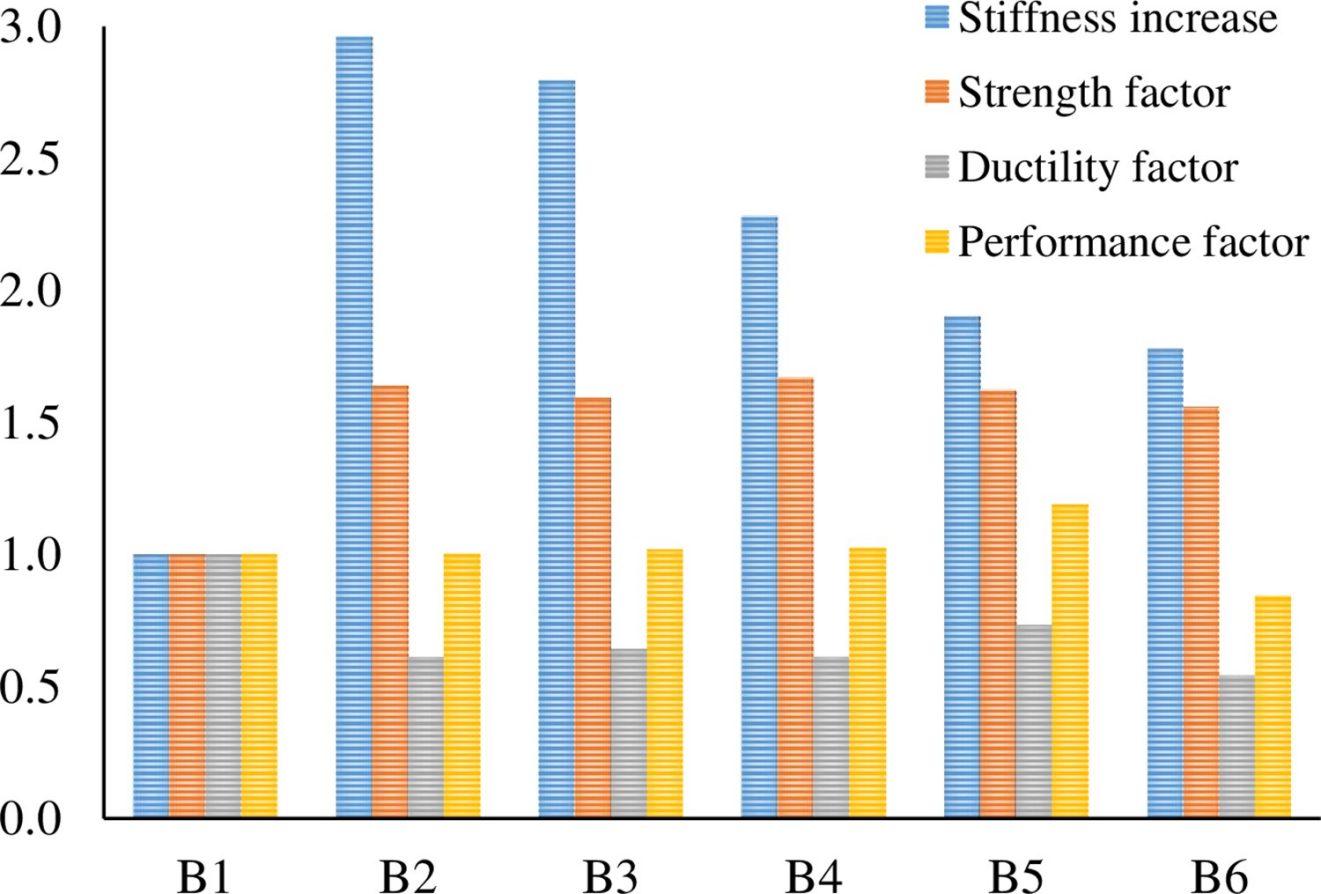
Stiffness increase, strength factor, ductility factor, and performance factor.

Table 8 and Fig 17 illustrate the toughness, ductility, performance factor, and initial stiffness of the R.C. beams. Toughness is the area under the load-deflection curve up to failure. Specimen B1 gives the highest area under the curve, which means it has a ductile behavior so that energy could be dissipated. On the other hand, the strengthened beams B2-B6 provide approximately similar toughness and dissipate energy less than B1 because the failure was controlled by CFRP debonding.

**Table 8:** Strength factor, stiffness, toughness, ductility factor, and performance factor.

RC beams	Strength (kN)	Strength factor (SF)	Stiffness (kN/mm)	Increase of stiffness	Toughness (kN.mm)	Ductility	Ductility factor (DF)	Performance Factor (PF)
**B1**	61.6	1.00	10.3	1.00	2541.6	3.7	1.00	1.00
**B2**	101.0	1.64	30.5	2.96	1538.9	2.26	0.61	1.00
**B3**	98.1	1.59	28.8	2.80	1172.6	2.37	0.64	1.02
**B4**	102.9	1.67	23.5	2.28	1448	2.27	0.61	1.02
**B5**	100.0	1.62	19.6	1.90	1811.3	2.71	0.73	1.19
**B6**	96.0	1.56	18.3	1.78	1325.5	2	0.54	0.84

Strength factor (S.F.): strength/ strength of the control beam.

Ductility factor (D.F.): ductility/ ductility of the control beam.

Performance factor (P.F.): SF X DF.

In addition, B1 has more ductility than the strengthened R.C. beams, which was apparent during the loading test. Ductility is the ultimate deflection divided by deflection at the yield of the steel bars. The ductility of beams B2-B6 was reduced by 39%, 36%, 39%, 27%, and 46%, respectively. Furthermore, B2, B3, and B4 have similar ductility. Also, B5 has the highest ductility of 2.71 among all the strengthened R.C. beams. However, specimen B6, with the highest pre-damage level, has the lowest ductility of 2.

Bonding CFRP to the concrete surface increases the initial stiffness of the strengthened R.C. beams because CFRP has a high modulus of elasticity. Specimen B2 has the highest initial stiffness of 30.5kN/mm; however, B1 has the lowest initial stiffness of 10.3kN/mm. B3, which is pre-damaged up to before steel bar yield, has nearly similar stiffness (28.8kN/mm) to B2. The initial stiffness of B2, B3, B4, B5, and B6 are increased by 196%, 180%, 128%, 90%, and 78% compared to the initial stiffness of B1, respectively.

**Fig 18:**
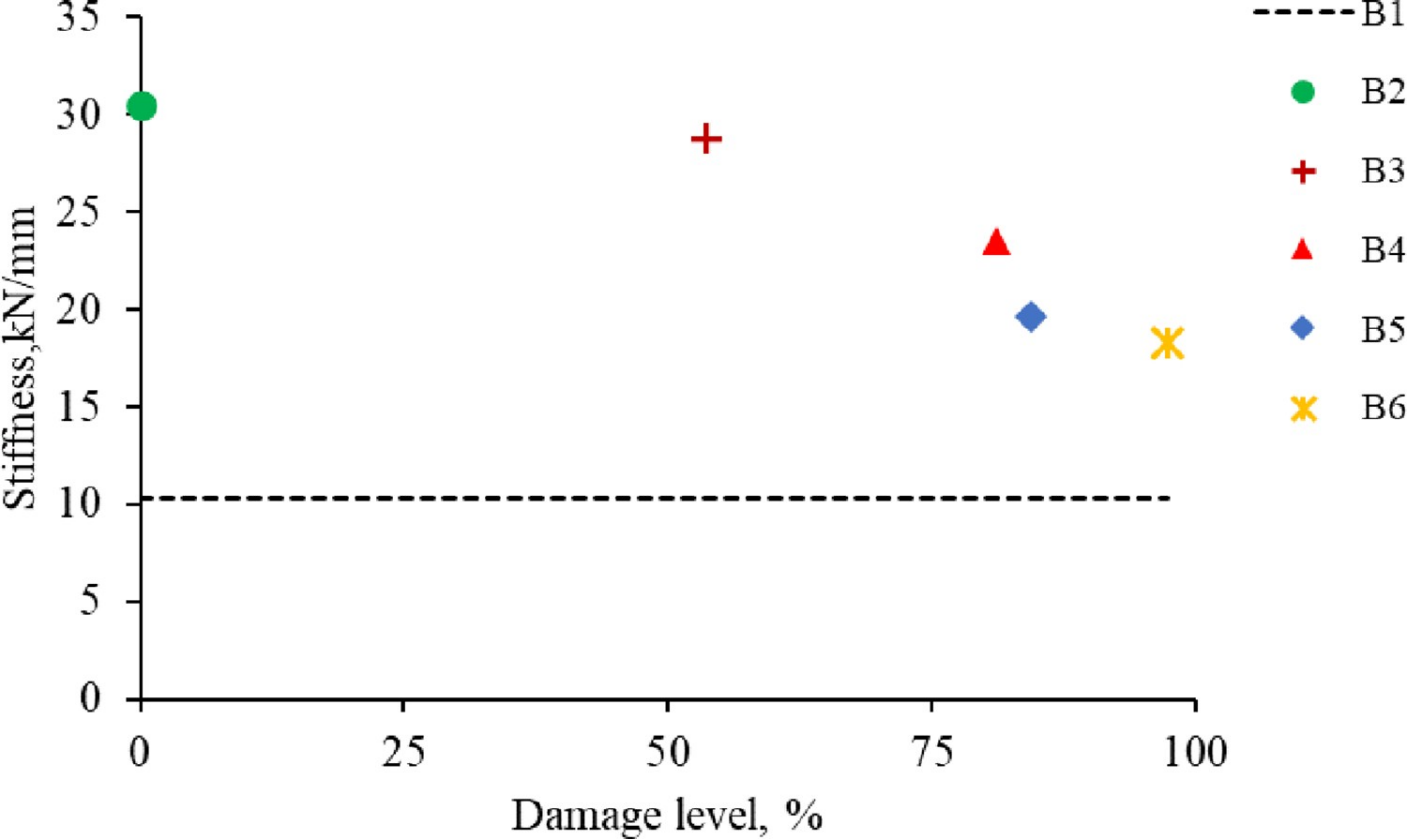
Stiffness and damage levels.

Increasing the damage level reduces the initial stiffness of the strengthened R.C. beams, as shown in Fig 18, because the concrete is cracked, and the steel bar is stressed before CFRP bonding. B3, B4, B5, and B6 reduced their initial stiffness by 5.6%, 23%, 36%, and 40%, respectively, as compared to B2.

The performance factor is the product of the ductility factor and strength factor. All the R.C. beams have approximately similar performance factors greater than one except for B6, which has the lowest performance factor, less than one.

## 4. Comparison with ACI 440

FRP strengthening systems should be designed to resist tensile forces while maintaining strain compatibility between the FRP and the concrete substrate. Design recommendations presented in ACI committee 440 [26] are based on the traditional reinforced concrete design principles stated in the requirements of ACI 318–05 and knowledge of the specific mechanical behavior of FRP reinforcement.

The constituent materials, fibers, and resins of an FRP system affect its durability and resistance to environmental exposure. Table 9 illustrates environmental reduction factors for the various FRP systems and exposure conditions. If the FRP system is located in a relatively non-threatening environment, such as indoors, the reduction factor is closer to unity. A lower reduction factor should be used if the FRP system is located in an aggressive environment where prolonged exposure to high humidity, freezing-and-thawing cycles, saltwater, or alkalinity is expected.

**Table 9:** Environmental reduction factor for various FRP systems and exposure conditions [26].

Exposure conditions	Fiber type	Environemntal reduction factor CE
**Interior exposure**	Carbon	0.95
	Glass	0.75
	Aramid	0.85
**Exterior exposure (bridges, piers, and unenclosed parking garages)**	Carbon	0.85
	Glass	0.65
	Aramid	0.75
**Aggressive environment (chemical plants and wastewater treatment plants)**	Carbon	0.85
	Glass	0.50
	Aramid	0.70

Eqs 1 through 3 give the tensile properties that should be used in all design equations. The design ultimate tensile strength and rupture strain should be reduced for environmental exposure conditions.

Because FRP materials are linear elastic until failure, Hooke’s law can determine the design modulus of elasticity for unidirectional FRP. The expression for the modulus of elasticity, given in 3, recognizes that the modulus of elasticity is typically unaffected by environmental conditions.

Other design guides such as [24] is required that the environmental conditions must be taken into account from the start of the design process. It obligates that design values of the FRP composite systems should account for reductions due to environmental exposure. However, specific values of such reductions are not given it suggests being supplied by the manufacturer. It is also suggested that in high alkalinity and high moisture or relative humidity environments CFRP should be used for strengthening of R.C. structures.

The Concrete Society Technical Report 55 [25] does not account for environmental reduction factor in the design process. However, partial safety factors for strength and modulus of elasticity at the ultimate limit state and for type of system (and method of application or manufacture) are applied to the characteristic mechanical properties of the FRP.


$$f_{fu}=C_{E}\;{f^{*}}_{fu}$$ 1

$$\varepsilon_{fu}=C_{E}\;{\varepsilon^{*}}_{fu}$$ 2

Ef=ffuεfu 3

Where; f*_fu_ and ε*_fu_ are ultimate tensile strength and rupture strain of CFRP from the manufacture datasheet, respectively. *f*_*fu*_ and *ε*_*fu*_, are ultimate tensile strength and strain of CFRP after environmental reduction, respectively.

Most of the externally bonded FRP system fails in FRP debonding or cover delamination. To prevent such debonding failure mode, the effective strain in FRP reinforcement should be limited to the strain level at which debonding may occur, ε_fd_, as defined in Eq 4.


$$\varepsilon_{fd}=0.41\sqrt{\frac{{f^{\prime }}_{c}}{n\;E_{f}t_{f}}}\leq 0.9\varepsilon_{fu}$$ 4

Where; *f*′_*c*_ is concrete compressive strength, *ε*_*fd*_ is designed strain, *E*_*f*_ is modulus of elasticity, *t*_*f*_ is thickness, and *n* is number of plies of CFRP.

**Fig 19:**
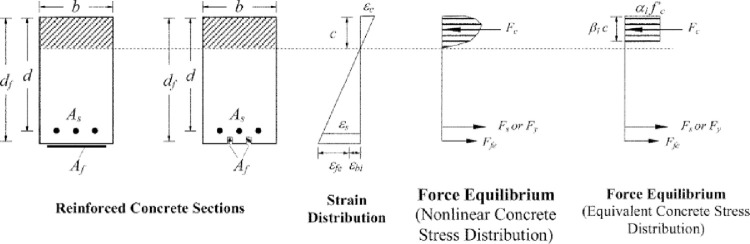
Internal strain and stress distribution for a rectangular section under flexure at ultimate limit state [26].

The calculation procedure described in [26] illustrates a trial-and-error method. The trial-and-error process involves selecting an assumed depth to the neutral axis c, calculating the strain level in each material using strain compatibility, calculating the associated stress level in each material, and checking internal force equilibrium. If the internal force resultants do not equilibrate, the depth to the neutral axis should be revised and the procedure repeated. Fig 19 shows strain compatibility, stress distribution, and forces equilibrium for a rectangular section strengthened with FRP at the ultimate limit state.

Eqs 5 and 6 calculate the effective strain and effective stress in the FRP, respectively. In Eq 5, ε_bi_ is the initial substrate strain, equal to zero in our case because the R.C. beams were unloaded and upturned before applying the CFRP sheets. If the left term of the equation controls, concrete crushing controls flexural failure of the section. However, if the right term of the equation controls, FRP failure (rupture or debonding) controls flexural failure of the section.


$$\varepsilon_{fe}=\varepsilon_{cu}\;\left[\frac{d_{f-C}}{c}\right]-\varepsilon_{bi}\leq \varepsilon_{fd}$$ 5

$$f_{fe}=E_{f}\varepsilon_{fe}$$ 6

Where; *f*_*fe*_, *ε*_*fe*_ and *d*_*f*_ are effective strength, strain, and depth of CFRP, respectively, *C* is the depth of neutral axis, *ε*_*cu*_ is ultimate strain of concrete, and *ε*_*bi*_ is initial substrate strain.

The strain and stress in the tensile steel bars are calculated using Eqs 7 and 8, respectively. The neutral axis is calculated using Eq 9.

The procedure is repeated until the equilibrium of the internal forces and strain compatibility are satisfied.


$$\varepsilon_{s}=(\varepsilon_{fe}+\varepsilon_{bi})\;\left[\frac{d-c}{d_{f}-c}\right]$$ 7

$$f_{s}=E_{s}\;\varepsilon_{s}\leq f_{y}$$ 8

$$c=\frac{A_{s}f_{s}+A_{f}f_{fe}}{\alpha_{1}{f^{\prime }}_{c}\beta_{1}b}$$ 9

Where; *ε*_*s*_, *f*_*s*_, *E*_*s*_, and *f*_*y*_ are strain, stress, and modulus of elasticity, and yield strength of steel reinforcement, respectively. *d* is effective depth of the section, *A*_*s*_ and *A*_*f*_ are area of steel and CFRP reinforcement, respectively. b is the width of the cross-section and α1 and β1 are the values associated with the Whitney stress block (α1 = 0.85 and β1 from ACI 318–05).

The nominal bending strength of the section with FRP external reinforcement is computed from Eq 10.


$$M_{n}=A_{S}f_{s}\left[d-\frac{\beta_{1}c}{2}\right]+\psi_{f}A_{f}f_{fe}\left[d_{f}-\frac{\beta_{1}c}{2}\right]$$ 10

A reduction factor for FRP, ψf, is applied to the flexural strength contribution of the FRP reinforcement. Proposed value of ψf is 0.85. The strength reduction factor, ψf, is used to improve the reliability of strength prediction and accounts for the different failure modes observed for FRP-strengthened members (delamination of FRP reinforcement).

**Fig 20:**
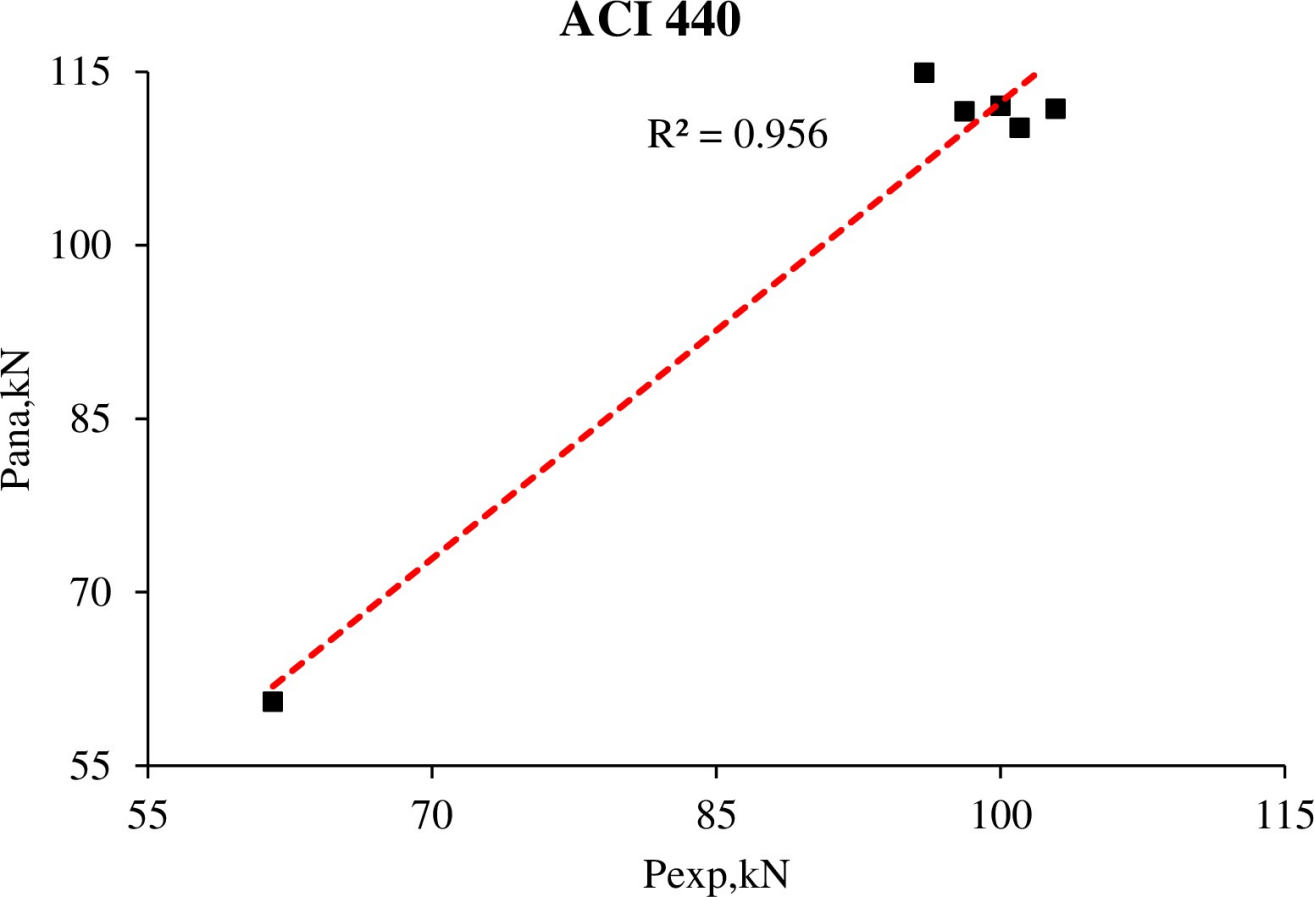
Relation between experimental and ACI 440 ultimate load capacity.

Table 10 and Fig 20 illustrate the comparison between the ultimate analytical load obtained from [26] and the experimental ultimate load capacity. It can be concluded that ACI 440.2R predicts the ultimate load capacity marvelously for externally bonded FRP beams compared to the practical ultimate load capacity.

**Table 10:** Experimental and analytical comparison.

R.C. beams	Experimental		ACI 440		P_ana_/P_exp_
	P_exp,_ (kN)	mode of failure	P_ana_, (kN)	mode of failure	
**B1**	61.6	C.C	60.5	C.C	0.98
**B2**	101.0	F.D	110.2	F.D	1.09
**B3**	98.1	F.D	111.6	F.D	1.14
**B4**	102.9	F.D	111.8	F.D	1.09
**B5**	100.0	F.D	112.1	F.D	1.12
**B6**	96.0	F.D	114.9	F.D	1.20

## 5. FEM analysis

The R.C. beams were modeled for finite element analysis using ABAQUS CAE, and the results are calibrated based on the experimental results. Four solid plates with (60mm x 20mm x 200mm) dimensions were used to model the loading plates and the supports. The plates were modeled as pure elastic behavior using (E = 200GPa).

### 5.1. Constitutive material

The material properties presented in section ‎2.2‎ and 2.3 were used to model the concrete and the steel reinforcement, respectively. The concrete Poisson’s ratio was taken as 0.2 for all the R.C. beams. Because CFRP is orientated in a uniaxial direction only, the CFRP sheet was modeled as linear elastic isotropic up to failure with the same data obtained from the manufacture datasheet presented in section ‎2.4. and assumed Poisson’s ratio (0.3). Furthermore, the stress-strain curve of the steel reinforcement obtained from the experimental test, as presented in section ‎2.3, was used to model the steel reinforcement properties with Poisson’s ratio equal to 0.3.

The concrete constitutive models implemented in ABAQUS include the Concrete Smeared Cracking model and the Concrete Damaged Plasticity (CDP) model. Since CDP is a powerful tool to model the plastic behavior of concrete, in this study only CDP is utilized to model the concrete behavior. CDP includes plasticity, compressive behavior, and tensile behavior.

Table 11 illustrates the final value of the parameters used to model the plasticity of the concrete after verification with the experimental results.

**Table 11:** Concrete damaged plasticity parameters.

Dilation Angle	Eccentricity	fb0/fc0	K	Viscosity Parameter
40	0.1	1.16	0.6667	0.0078

**Fig 21:**
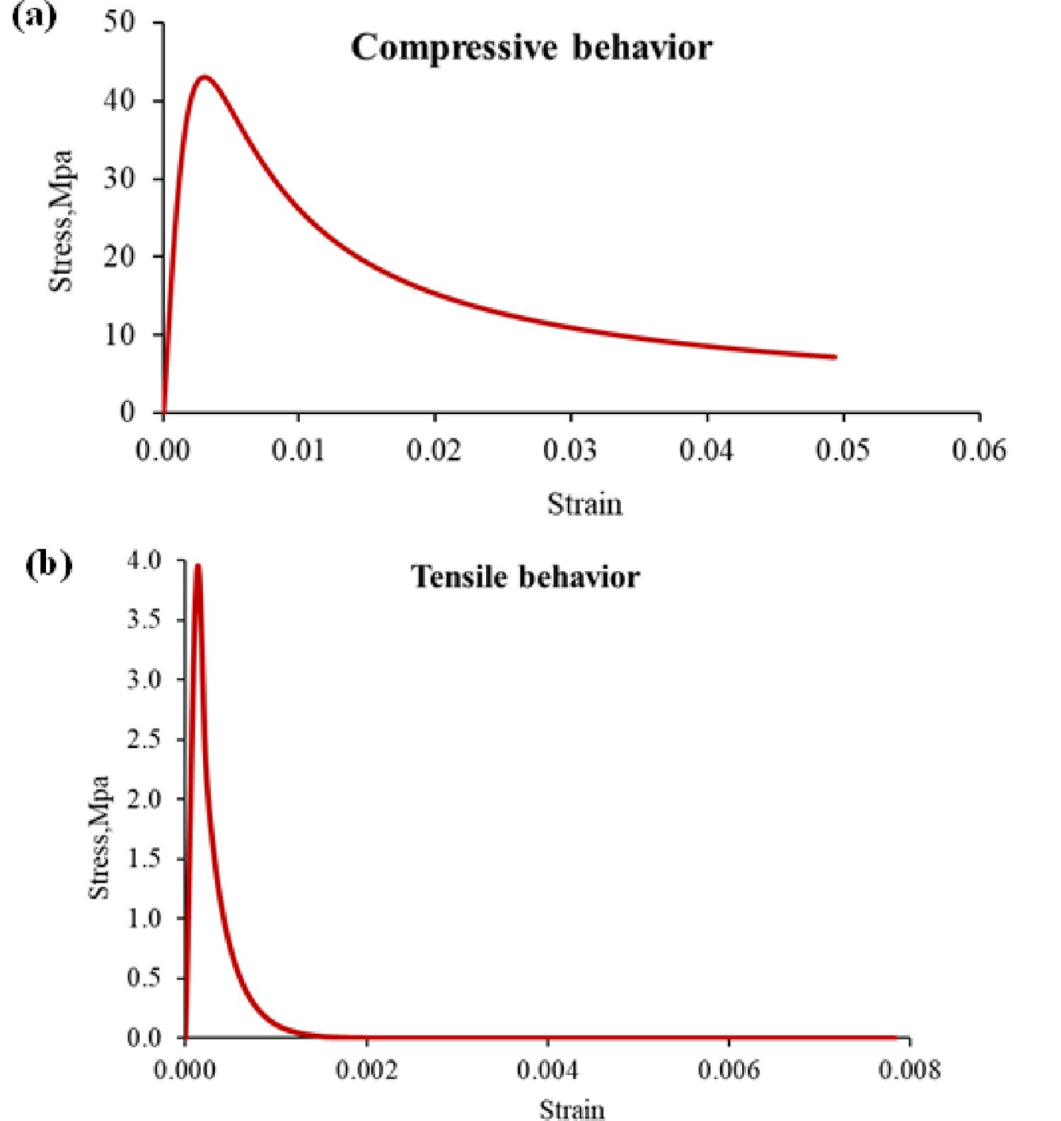
Concrete behavior: (a) compressive behavior, (b) tensile behavior.


Because the descending part of the compressive stress-strain curve could not be achieved experimentally, Chin and Mansur [27] model is used to model the compressive behavior of the concrete. Eqs 11 through 13 and Fig 21A present the concrete compressive behavior. The model is chosen based on the compatibility of the ascending part of the experimental stress-strain curve with the model.

$$f_{c}={f^{\prime }}_{c}\;\left[\frac{k_{1}\beta (\frac{\varepsilon }{\varepsilon_{0}})}{k_{1}\beta -1+{\left(\frac{\varepsilon }{\varepsilon_{0}}\right)}^{k_{2}\beta }}\right]$$ 11

Where;

$$k_{1}={\left(\frac{50}{{f^{\prime }}_{c}}\right)}^{3.0},\;k_{2}={\left(\frac{50}{{f^{\prime }}_{c}}\right)}^{1.3}$$ 12

β=11−(f′cε0Eit) 13

*ε*_0_ and *E*_*it*_ are initial strain and modulus of elasticity of concrete, respectively. *f*_*c*_ and *ε* strength and strain of concrete, respectively.

Furthermore, Sneed [28] model is used to model the tensile behavior of the concrete. Eq 14 and Fig 21B illustrate the tensile behavior of the concrete.


$$\sigma =f_{t}{\left(\frac{\varepsilon_{t}}{\varepsilon }\right)}^{\left(0.7+1000\varepsilon \right)},\;\varepsilon_{t}=\frac{f_{t}}{E_{c}}$$ 14

Where; *f*_*t*_ and *ε*_*t*_ tensile strength and strain of concrete, respectively. *σ* and *ε* tensile stress and strain of concrete, respectively. *E*_*c*_ modulus of elasticity of concrete.

### 5.2. Meshing

The R.C. beams had been optimized using mesh sensitivity and chosen meshing size of 20mm was found to best approximate the overall structural response of the examined beams and be less time-consuming.

An 8-node linear brick, reduced integration, hourglass control (C3D8R) element type is assigned to the concrete and loading plates. Furthermore, a 2-node linear beam in space (B31) element type is set to the reinforcement cage. Finally, a 4-node doubly curved thin or thick shell, reduced integration, hourglass control, finite membrane strains (S4R) are assigned to the CFRP sheet.

### 5.3. Interactions

The interaction between the concrete and steel reinforcement and the R.C. beam and the loading plates should be defined in ABAQUS. A perfect bond between the concrete and steel reinforcement was assumed using the embedded region. In the embedded region, the concrete is the host, and the steel reinforcement is the embedded region. The perfect bond assumption may lead to higher stiffness of the model in the load-deflection curve response than the experimental. Furthermore, a perfect bond between the loading plates and the R.C. beam is assumed using Tie contact. The R.C. beam is the master surface; however, the loading plates are the slave surface.

### 5.4. CFRP and concrete interface

The model for the interface between FRP and concrete is of essential importance. A perfect bond model and cohesive models were evaluated for describing the concrete-FRP interface. The ultimate load and stiffness were overestimated with a perfect bond between the FRP and concrete compared to experimental results. The reason for this is that degradation in the bond cannot be captured in this type of model, and it implies that a perfect bond model is not suitable in a study focusing on debonding.

**Fig 22:**
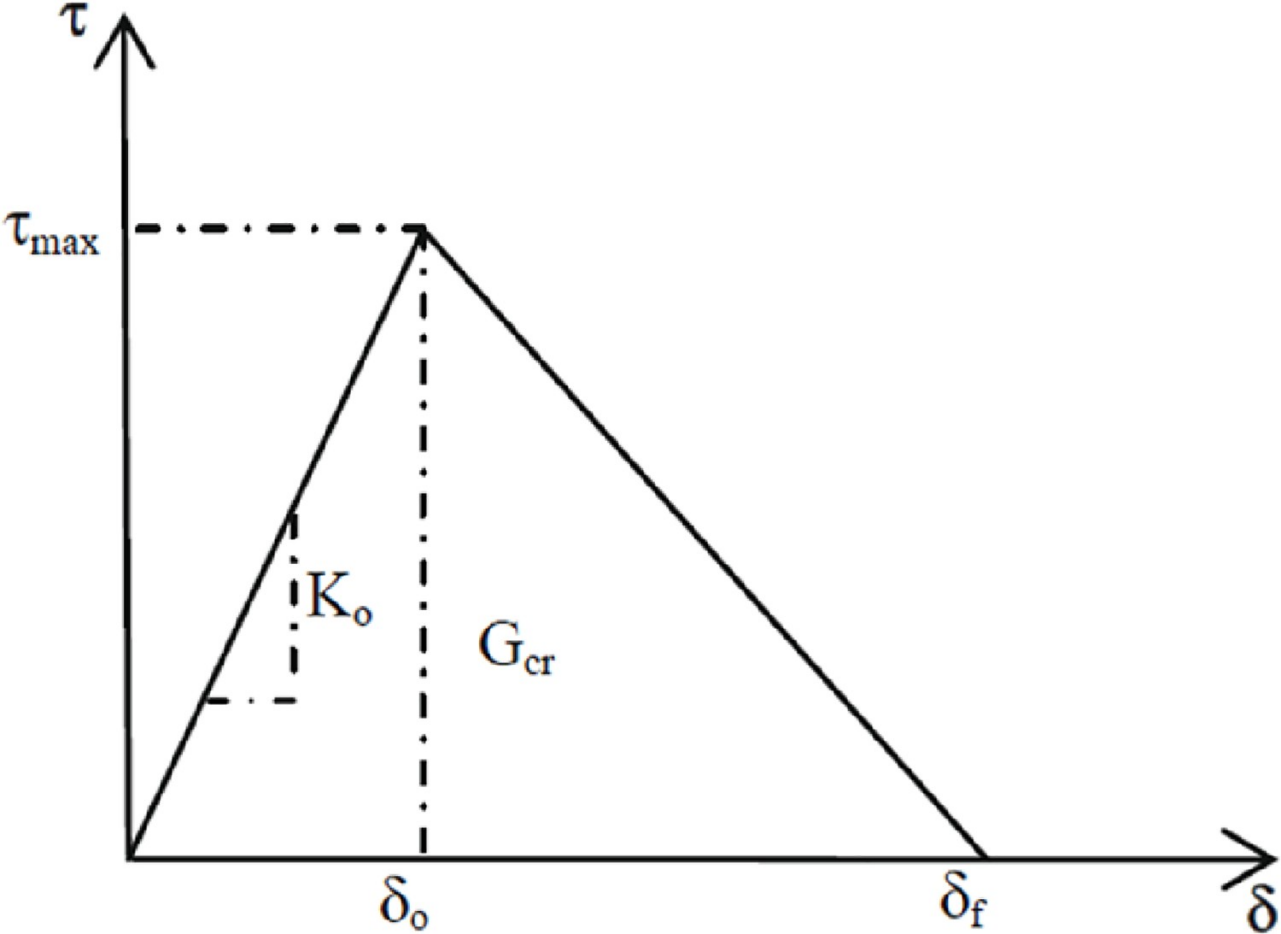
Bond-slip curve, bilinear model [29].

The cohesive model available in ABAQUS is a better choice for representing the interface behavior. The cohesive model defines surfaces of separation and describes their interaction by defining a relative displacement at each contact point. The definition of the model is characterized by the parameters, initial stiffness, shear strength, fracture energy that equals the area under the traction–displacement curve, and the curve shape of the bond-slip model. Fig 22 shows a graphic interpretation of a simple bilinear traction–separation law written in terms of the effective traction τ and effective opening displacement δ, as recommended by [29]. In previous models, the input data was only related to the concrete properties, and it was considered necessary also to include the adhesive properties.

The following relations 15 through 17 for initial stiffness, shear strength, and fracture energy, as a function of the adhesive and concrete properties, were recommended by [29]:

$$K_{nn}=\frac{1}{\frac{t_{c}}{E_{c}}+\frac{t_{epoxy}}{E_{epoxy}}}$$ 15

Where; K_nn_ is normal stiffness from peeling off test, t_c_ is the concrete thickness = 5 mm, t_epoxy_ is the epoxy or adhesive thickness = 1 mm, E_c_ = Young’s modulus of concrete, and E_epoxy_ = Young’s modulus of adhesive. The approximate value of K_nn_ = 1700MPa.


$$K_{ss}=K_{tt}=\frac{1}{\frac{t_{c}}{G_{c}}+\frac{t_{epoxy}}{G_{epoxy}}}$$ 16

Where; K_ss_ = K_tt_ = shear stiffness from lap joint test, G_c_ is Shear modulus of concrete = 10800 MPa, G_epoxy_ is Shear modulus of epoxy or adhesive = 665MPa.


$$\tau_{max}=1.5\;x\;f_{ct}\;x\sqrt{\left[2.25-\frac{b_{f}}{b_{c}}]/[1.25+\frac{b_{f}}{b_{c}}\right]}$$ 17

Where; f_ct_ = Tensile strength of concrete, MPa, b_f_ = FRP plate width, mm, b_c_ = Concrete width, mm.

The damage initiation was assumed to occur when a quadratic traction function involving the nominal stress ratios reached value one.

Interface damage evolution has been expressed in terms of energy release. The description of this model is available in the ABAQUS material library [30]. The dependence of the fracture energy on the mode mix was defined based on the Benzaggah–Kenane fracture criterion. Benzaggah–Kenane fracture criterion is particularly useful when the critical fracture energies during deformation are the same together with the first and second shear directions.

In order to find the values of initial stiffness, shear strength, and fracture energy that gave the best fit, simulations were performed, and the results were compared with experimental results. The shear strength has more effect on the debonding failure load and load-deflection curve than the other parameters. Table 12 presents the input value of the cohesive model.

**Table 12:** Parameters of the cohesive contact between CFRP and concrete substrate.

Cohesive behavior		Damage					
		Initiation		Evolution		Stabilization	
**K**_**nn**_	1360 MPa	**Normal**	Tensile strength, MPa	**Normal Fracture energy**	0.09 mJ/mm2	**viscosity coefficient**	1*x*10^−5^
**K**_**ss**_	525 MPa	**Shear-1**	0.7 MPa	**1st shear fracture energy**	0.9 mJ/mm2		
**K**_**tt**_	525 MPa	**Shear-2**	0.7 MPa	**2nd shear fracture energy**	0.9 mJ/mm2		

### 5.5. Loading and boundary condition

The pre-loading scheme distinguished in the laboratory test was also considered in the numerical calculations by creating a pre-defined field. Loading and unloading simulations, as the experiment, are conducted on the R.C. beam models before CFRP strengthening to obtain pre-loading scheme. Then the pre-loading is added to the CFRP strengthened R.C. beams through the pre-defined field by importing and assigning the stress from the output file of the loading and unloading simulation to the steel reinforcement and concrete beam elements separately at the initial stage of the analysis.

An incremental iterative procedure was employed to obtain a solution for the analyzed structures and loading programs. Two static point loads were applied at the loading plates corresponding to the experimental location using displacement control. The displacement increments were implemented as a smooth step in the loading amplitude.

The boundary condition of the supporting plates was specified at the appropriate nodes as a simply supported beam. One plate has restrained the displacement in the X and Y direction representing pin support, and the other plate has restrained the displacement only in the Y direction representing roller support.

When performing a nonlinear analysis, convergence difficulties may occur, especially when cracks start to initiate. To prevent convergence issues, dynamic explicit integration was used to perform nonlinear analysis.

### 5.6. F.E. Results

**Fig 23:**
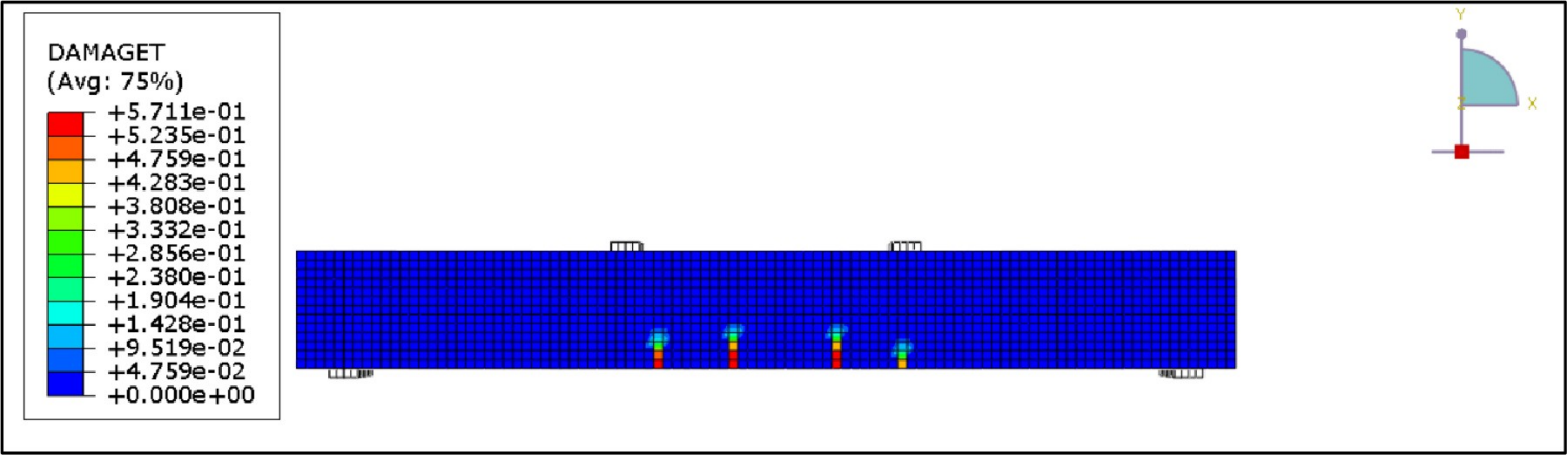
First crack of beam B1 (control beam).

Fig 23 shows the first crack initiation of the control beam, B1. The first crack occurred at the load of 18.3kN at the time step of 0.1 seconds, which is 87% of the load obtained in the experiment.

**Fig 24:**
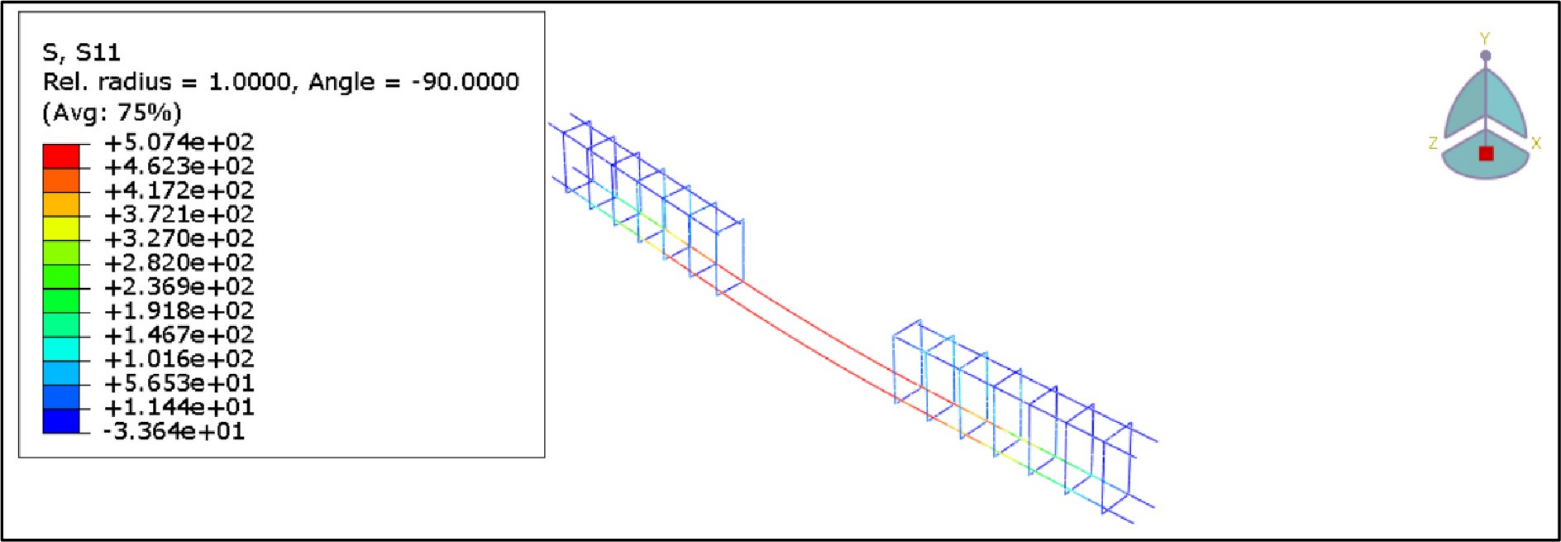
Stress in the reinforcement steel for the B1.

**Fig 25:**
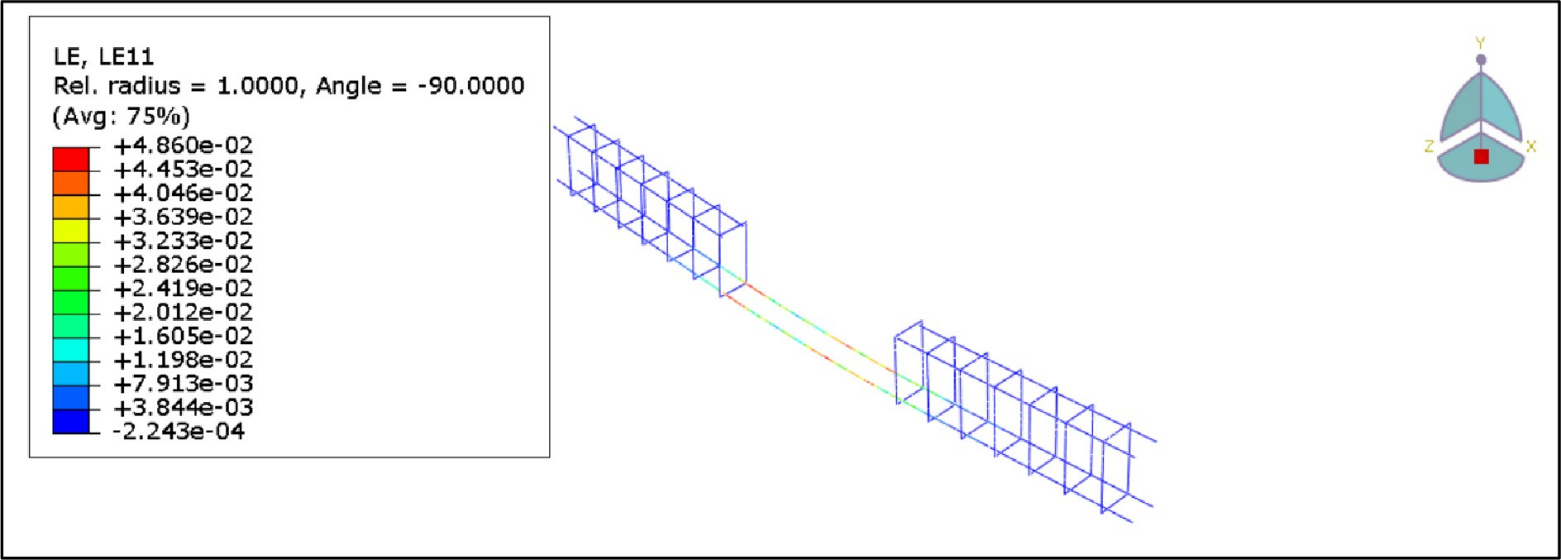
Strain in the reinforcement steel for the B1.

Furthermore, Figs 24 and 25 show axial stress and axial strain in the steel reinforcement at failure, respectively. It can be seen that the steel reinforcement reaches yielding before concrete crushing occurs.

**Fig 26:**
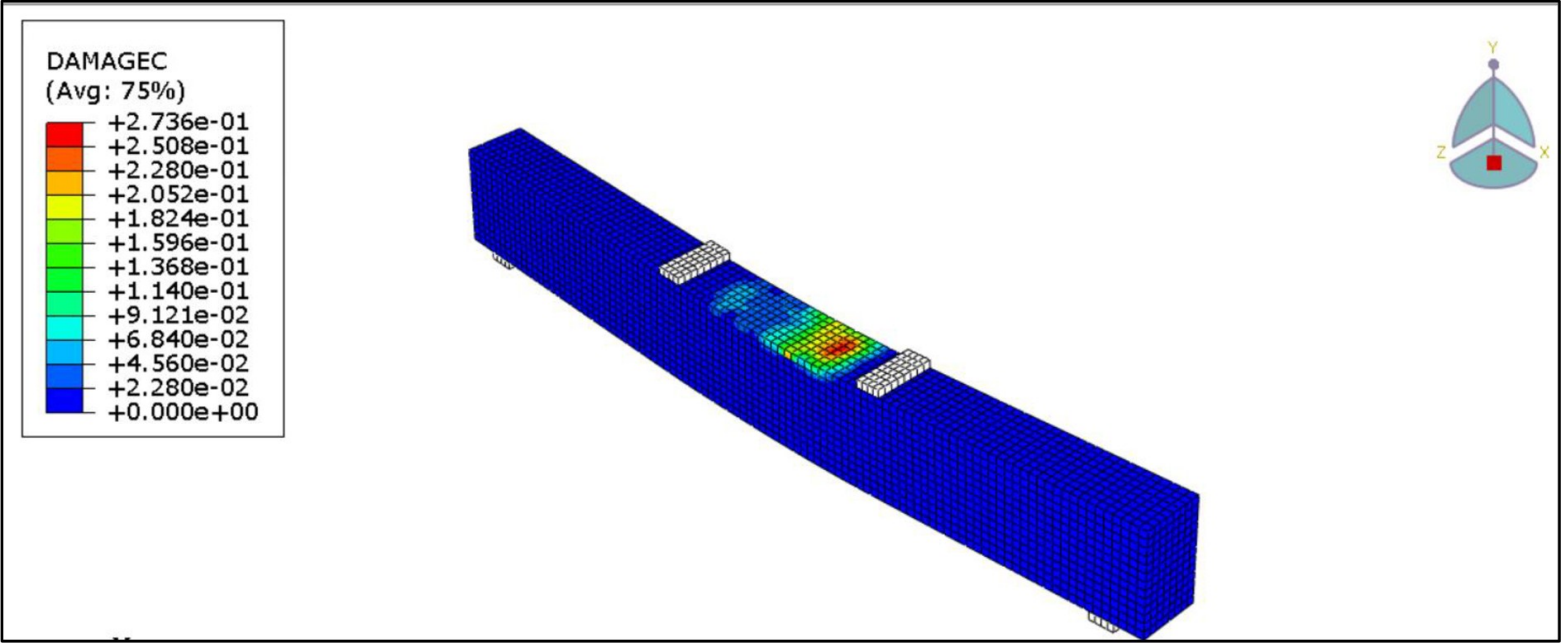
Crack patterns for the B1.

**Fig 27:**
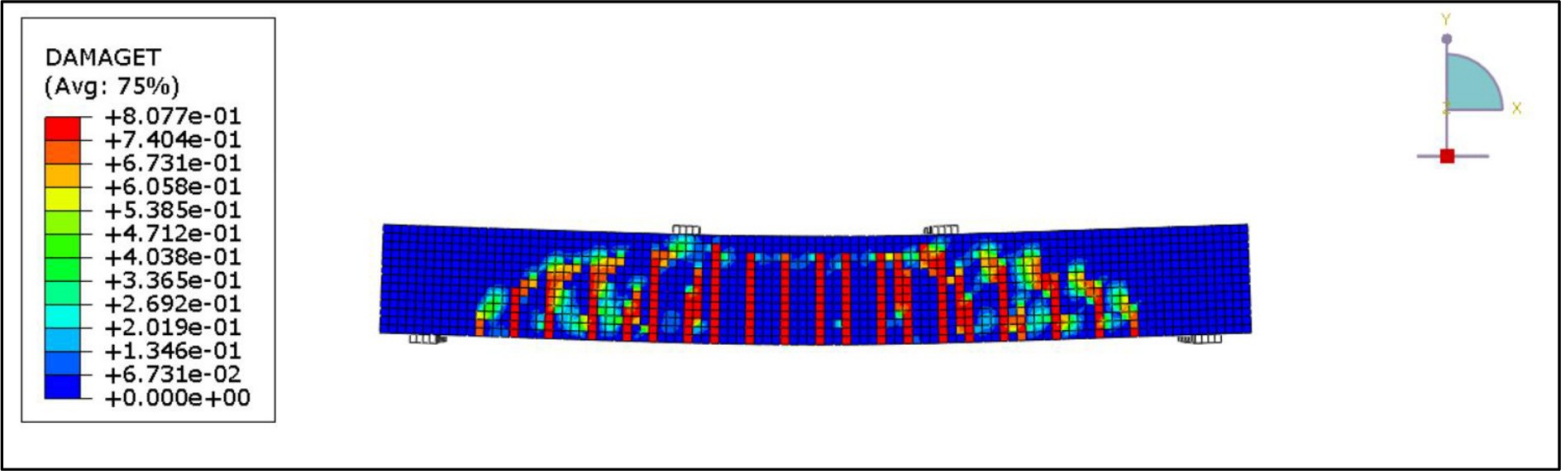
Concrete compression failure in the B1.

Fig 26 shows the flexure crack propagation in the control beam, B1. Also, Fig 27 shows the concrete compression failure of the control beam. The crack patterns and concrete crushing are all well agreed with the experiment.

**Fig 28:**
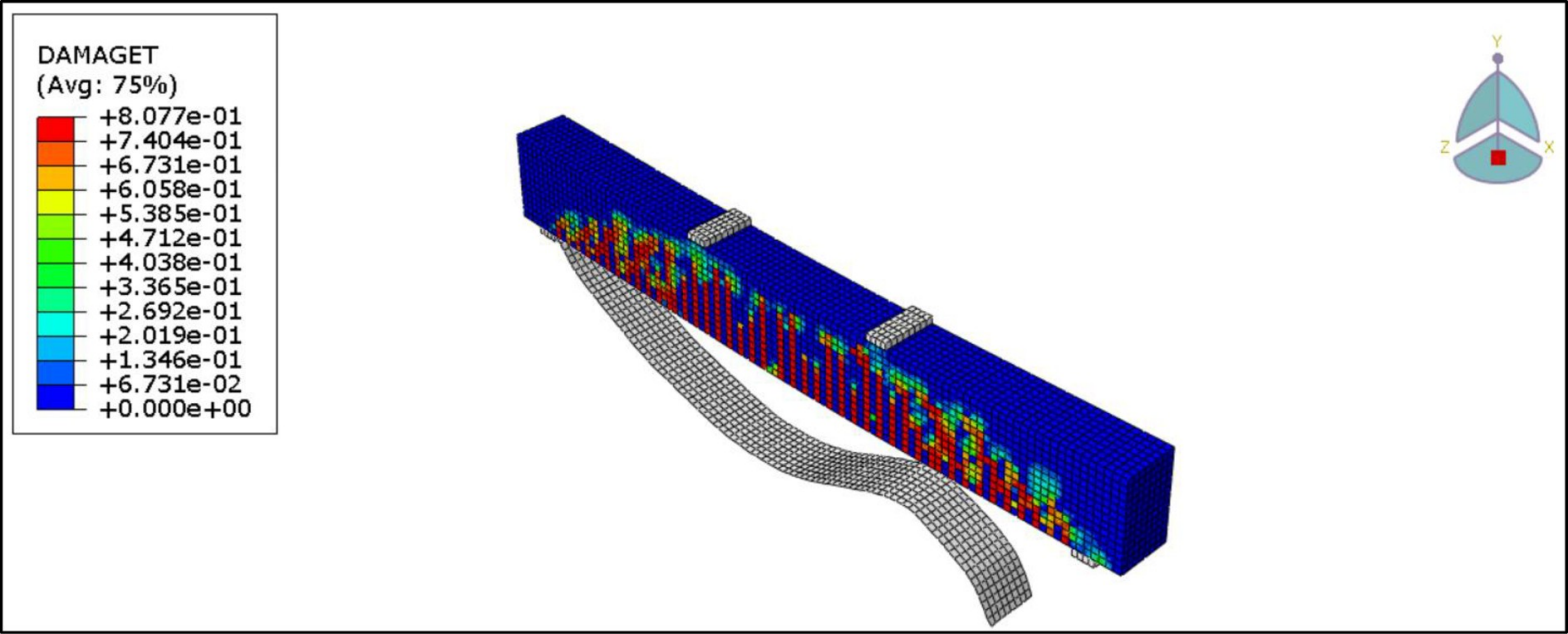
CFRP Debonding for the R.C. beams.

Fig 28 presents the CFRP debonding failure of the strengthened R.C. beams B2-B6. The debonding failure of the CFRP sheet can be well captured using the cohesive interface.

**Fig 29:**
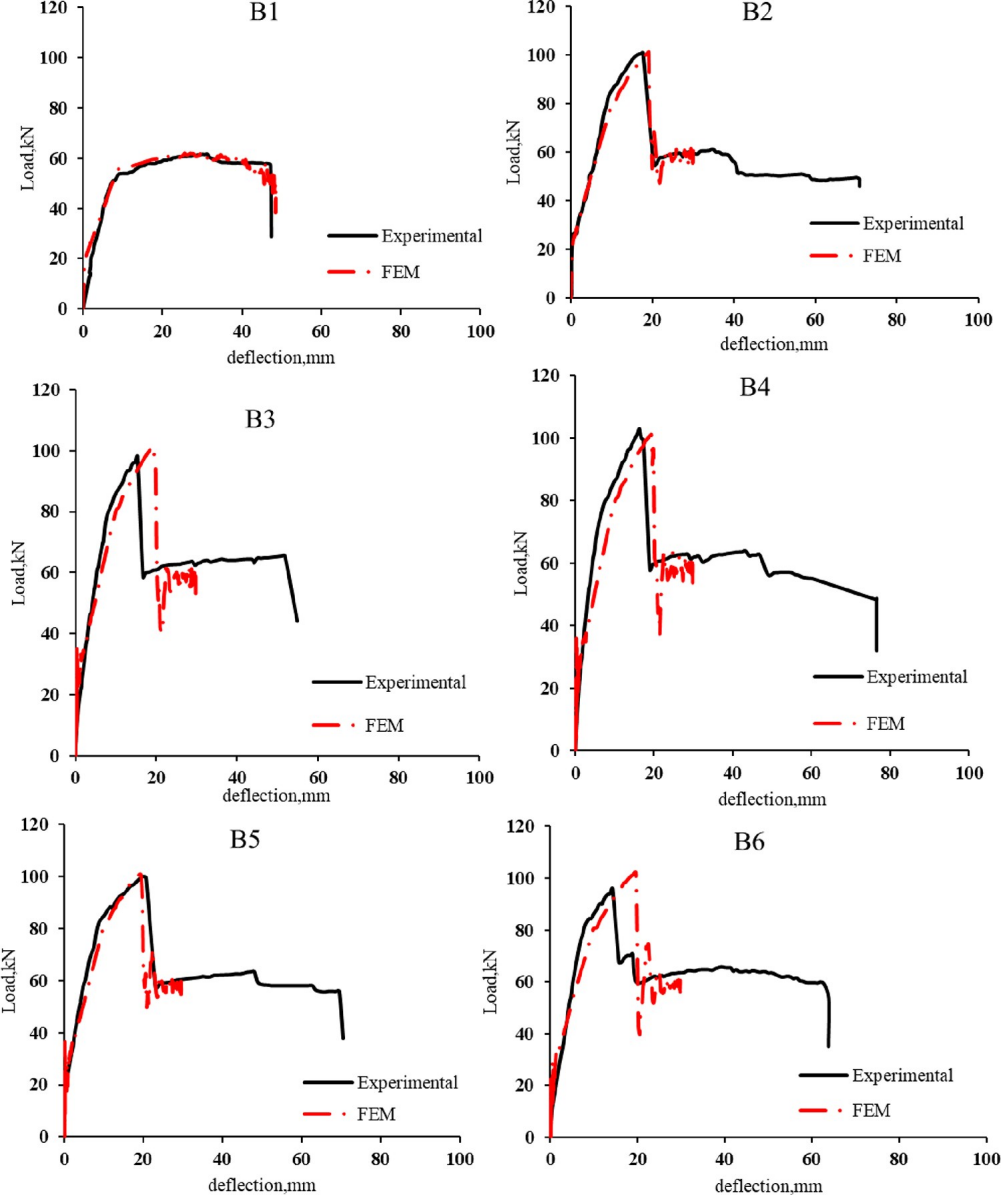
Experimental and F.E. load vs. mid-span deflection for the R.C. beams.

The load-deflection curves of the control beams and retrofitted R.C. beams obtained from the experiment and FEM analysis are illustrated in Fig 29. The FEM analysis predicts the beam to be a bit stiffer and stronger, probably because of the assumed perfect bond between concrete and reinforcement.

**Fig 30:**
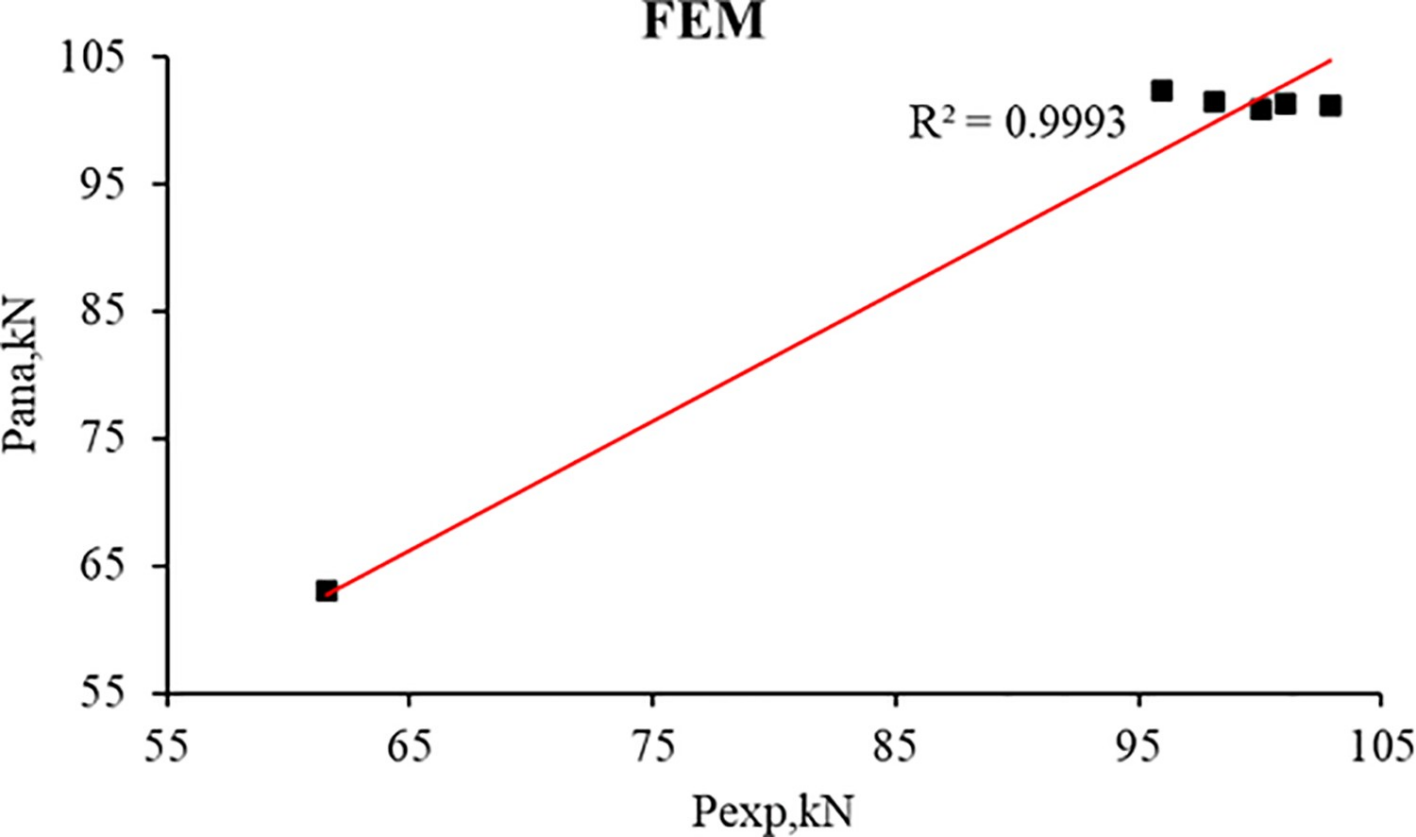
Relation between experimental and FEM load capacity of the R.C. beams.

**Fig 31:**
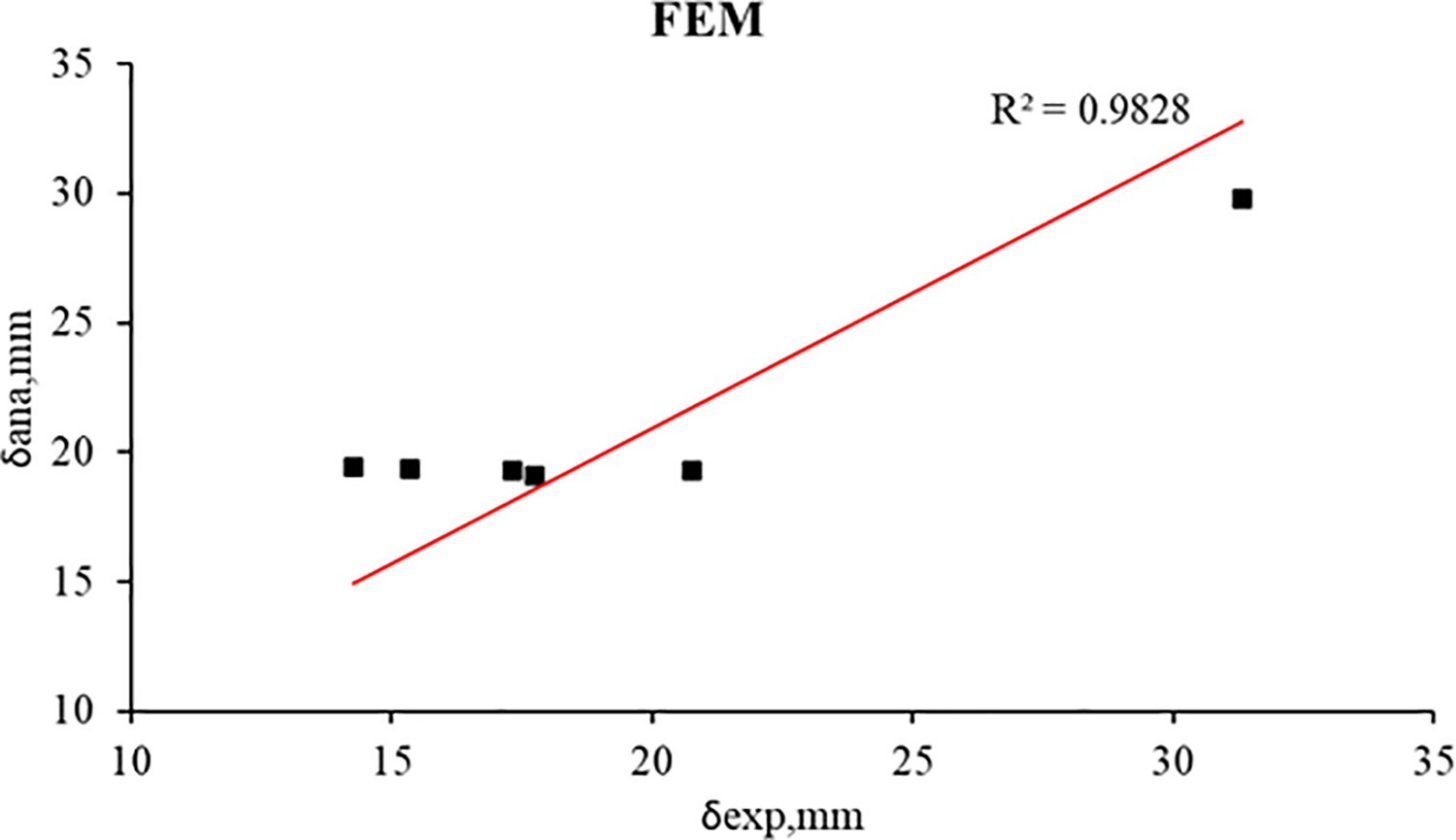
Relation between experimental and FEM mid-span deflection of the R.C. beams.

Table 13, Figs 30, and 31 present the comparison between experimental and analytical ultimate load and mid-span deflection. It can be concluded that there is a good agreement between FEM and experimental results for the control beams and strengthened R.C. beams. The good agreement indicates that the constitutive models used for concrete and reinforcement and the cohesive interface model can well capture fracture behavior.

**Table 13:** Experimental and FEM comparison of the R.C. beams.

R.C. beams	Experimental		FEM		P_ana_/P_exp_	δ_ana_/δ_exp_
	P_exp_ (kN)	mid-span deflection (δ), mm	P_ana_, (kN)	mid-span deflection (δ), mm		
**B1**	61.6	31.3	63.1	29.8	1.02	0.95
**B2**	101.0	17.7	101.4	19.1	1.00	1.08
**B3**	98.1	15.4	101.6	19.3	1.04	1.26
**B4**	102.9	17.3	101.1	19.3	0.98	1.12
**B5**	100.0	20.8	100.9	19.3	1.01	0.93
**B6**	96.0	14.3	102.3	19.4	1.07	1.36

## 6. Conclusion

The experimental and numerical investigations were carried out on six R.C. beams to study the influence of pre-damage loading on the load capacity and structural performance of CFRP strengthened R.C. beams. One beam is left as control beam, the other five beams were strengthened in flexure. Four R.C. beams are pre-damaged to different levels of strain in the steel bar before CFRP strengthening. The experimental results compared to the design guideline. Also, the FEM calibrated based on the experimental load-deflection response. The following conclusions can be drawn:

- The failure mode of the CFRP strengthened specimen was controlled by CFRP debonding followed by concrete crushing; however, the control beam failed in concrete crushing after yielding the steel bars, which is typical tension control failure according to ACI-318.
- The epoxy resin properties significantly influence the debonding failure because the epoxy resin ruptured in tension before CFRP debonding. So, improving the epoxy properties, predominantly tensile and flexure strength, enhances the bond strength and postpones the CFRP debonding.
- CFRP sheet restores and upgrades the strength and stiffness of the damaged R.C. beams; however, it reduces ductility and toughness. Also, CFRP application increases the first crack and yielding steel bars load by 87.4% and 34.4%, respectively.
- The pre-damage level does not influence the strength and ductility of the strengthened R.C. beams except for the highest damage levels, which experienced a slight decrease in load capacity and ductility. However, the initial stiffness decreases with increasing pre-damage levels by 40%.
- ACI 440.2R predicts the ultimate load capacity marvelously for externally bonded FRP beams compared to the experimental ultimate load capacity.
- It can be concluded that there is a good agreement between FEM and experimental results for the control beams and strengthened R.C. beams. The good agreement indicates that the constitutive models used for concrete and reinforcement and the cohesive interface model can well capture fracture behavior. However, The FEM analysis predicts the beam to be slightly stiffer and stronger, probably because of the assumed perfect bond between concrete and reinforcement. The developed FEM can be used for further parametric studies.

## Supporting information

S1 Data (XLSX)
